# Evaluating differences in uterine microbiome and inflammatory status at 1 month postpartum associated with metritis and antibiotic treatment

**DOI:** 10.3168/jds.2025-26403

**Published:** 2025-09-18

**Authors:** Joao G. N. Moraes, Tamara Gull, Aaron C. Ericsson, Monica O. Caldeira, Tim J. Evans, Scott E. Poock, Matthew C. Lucy

**Affiliations:** 1 Department of Animal and Food Sciences, Oklahoma State University, Stillwater, OK 74078; 2 Veterinary Medical Diagnostic Laboratory, College of Veterinary Medicine, University of Missouri, Columbia, MO 65201; 3 Division of Animal Sciences, University of Missouri, Columbia, MO 65201; 4 Veterinary Medical Extension and Continuing Education, College of Veterinary Medicine, University of Missouri, Columbia, MO 65201

**Keywords:** microbiome, uterus, antibiotics, metritis, inflammation

## Abstract

Ceftiofur treatment in cows with metritis enhances the likelihood of clinical cure but does not consistently improve subsequent pregnancy rates compared with healthy (nonmetritis) cows. We tested the hypothesis that ceftiofur treatment has no effect on uterine microbiota or endometrial inflammation at 1 mo postpartum in cows that were either healthy or diagnosed with metritis at 5 to 10 d postpartum. Cows diagnosed with metritis were matched with healthy cows in a 2 × 2 factorial design where they were either treated with an antibiotic (ceftiofur hydrochloride) or left untreated. Cows were slaughtered to collect the uterus at 1 mo postpartum, and the uterine microbiota was measured using bacteriology and 16S rRNA gene sequencing. The lumen of the uterus was examined visually for the presence of purulent material, and inflammation within the endometrium was assessed histologically. When bacteria were cultured from the lumen of the uterus at 1 mo postpartum, a greater number of species was isolated and a greater number of colonies formed from cows that were previously diagnosed with metritis compared with healthy cows. Antibiotic treatment administered at the time of disease diagnosis (5 to 10 d postpartum) had no effects on the abundance of culturable bacteria at 1 mo postpartum in healthy cows but reduced the number of culturable bacteria from the uterus of cows previously diagnosed with metritis. Based on 16S rRNA gene sequencing, cows originally diagnosed with metritis had an increased number of sequencing reads 1 mo postpartum than cows initially deemed healthy. Furthermore, antimicrobial treatment 5 to 10 d postpartum decreased the number of sequencing reads 1 mo postpartum irrespective of the initial disease diagnosis. The presence of purulent or metritis-like discharge in the uterine lumen 1 mo postpartum had a major effect on the uterine microbiota. Histopathological analysis revealed that the presence of purulent material in the uterus or signs of acute infection at 1 mo postpartum was associated with greater uterine inflammation compared with a clear uterine flush but minimally affected by treatment. Furthermore, antibiotic treatment had no main effect on α-diversity (Faith’s phylogenetic diversity or Pielou’s evenness), and no differentially abundant taxa were detected by analysis of composition of microbes. However, β-diversity analyses (unweighted UniFrac) showed a small but significant effect of treatment, with treated cows having lower within-group variability than untreated cows, suggesting a modest homogenizing effect on the uterine microbiota. A multinomial regression analysis revealed that the relative abundance of *Fusobacterium* was 145.45 times lower in ceftiofur-treated cows compared to untreated cows. This shift suggests a partial restoration of the microbial community in cows with metritis toward a profile resembling that of their healthy counterparts. Additionally, the analysis indicated that the uterine microbiota at 1 mo postpartum was collectively influenced by visual detection of purulent material, occurrence of metritis postpartum, antibiotic treatment, and estrus cyclicity. We rejected the hypothesis that early postpartum ceftiofur treatment did not affect uterine microbiota at 1 mo postpartum and concluded that ceftiofur treatment had a measurable effect on uterine microbiota and inflammation.

## INTRODUCTION

Approximately one-third of dairy cows will develop metritis (uterine infection) within 1 wk after calving (Wagener et al., 2017; Pérez-Báez et al., 2021; Garzon et al., 2022). Cows with metritis are at risk for developing a second uterine disease, endometritis (uterine inflammation with purulent material in the uterine lumen) that can be present at the time of breeding (LeBlanc et al., 2002; Wagener et al., 2017; Koyama et al., 2018). Both metritis and endometritis place the cow at risk for subfertility, evidenced by a longer postpartum interval to establish pregnancy (Moraes et al., 2021; LeBlanc, 2023; Bruinjé et al., 2024). Subfertile dairy cows are more likely to be culled from the herd because they either never become pregnant or their interval to pregnancy is not economically viable (Overton and Eicker, 2025). In addition to reducing fertility, metritis adversely affects cow productivity, including milk yield (Menta et al., 2024). The cumulative effects of lost milk production, subfertility, and increased culling for a single case of metritis cost the farm ~$500 (Pérez-Báez et al., 2021).

Cows with metritis in the United States are typically treated with the antibiotic ceftiofur because it increases the risk of clinical resolution of the disease (Chenault et al., 2004; McLaughlin et al., 2012; de Oliveira et al., 2020). Ceftiofur has the added benefit of no milk withholding because it does not pass into the milk (Chenault et al., 2004). Ceftiofur leads to a rapid (within 2 d) shift in the uterine microbiota, specifically targeting *Fusobacterium necrophorum* (Jeon et al., 2018, 2021). Although ceftiofur has been shown to improve the clinical cure rate of cows with metritis (Chenault et al., 2004; McLaughlin et al., 2012; de Oliveira et al., 2020), its effectiveness in reducing the time to pregnancy postpartum has yielded mixed results (de Oliveira et al., 2020; Menta et al., 2024). In a recent study by Pereira et al. (2025a), clinical cure (reduction in vaginal discharge score) clearly increased the risk of pregnancy by 300 DIM, but antimicrobial treatment (which is known to increase the risk of clinical cure) did not increase the risk of pregnancy (Pereira et al., 2025a). The mechanisms associated with the risk of clinical cure and the risk of pregnancy for ceftiofur-treated and untreated cows, therefore, may be different. Figueiredo et al. (2024) examined the uterine microbiome, clinical cure, and pregnancy outcomes following ceftiofur treatment. They found that neither reduced fertility nor clinical cure failure in treated metritis cows was explained by the uterine microbiome (Figueiredo et al., 2024). Other mechanisms that include the uterine metabolome (de Oliveira et al., 2023) and the extent of uterine inflammation at the time of treatment administration (Pereira et al., 2025b) may explain the association between clinical cure and pregnancy outcomes, which appears to be complex and independent of antibiotic treatment. We found that inflammation in the uterus at 1 mo postpartum reduced endometrial glandular development (Sellmer Ramos et al., 2023) and altered the endometrial transcriptome (Caldeira et al., 2025b). Understanding the uterine microbiome and its role in the resolution of uterine inflammation may provide the key to explaining the relationship between metritis, antibiotic treatment, clinical cure, and fertility postpartum.

We explored the effects of ceftiofur treatment on the uterine microbiome and inflammation in the endometrium at 1 mo postpartum in cows that were initially diagnosed with metritis or healthy and treated systemically with an antibiotic at 5 to 10 d postpartum. The first objective was to determine if ceftiofur treatment affected the uterine microbiome at 1 mo postpartum. The second objective was to determine if ceftiofur affected inflammation in the endometrium at 1 mo postpartum. The null hypothesis was that antibiotic-treated cows (either healthy or metritis) would have a microbiome and uterine inflammation like that of untreated cows at 1 mo postpartum. If true, then this lack of an effect could explain why ceftiofur treatment may increase clinical cure early postpartum but fails to increase pregnancy risk, as ceftiofur does not have a lasting effect on uterine microbiome or uterine inflammation.

## MATERIALS AND METHODS

Study procedures were approved by the University of Missouri Institutional Animal Care and Use Committee (protocol number 9635). Supplemental figures, tables, and datasets are available (see Notes).

### Experimental Design

The study enrolled 35 first-parity Holstein cows from 2 farms (University of Missouri Foremost Dairy Research Center [Columbia, MO; n = 6 cows, start enrollment October 2019 and end enrollment November 2019; 1 healthy untreated, 3 healthy treated, 1 metritis untreated, 1 metritis treated] and Ohlde Dairy [Lynn, KS; n = 29 cows, start enrollment November 2019 and end enrollment March 2020; 7 healthy untreated, 6 healthy treated, 8 metritis untreated, 8 metritis treated]). The 2 dairies were typical confinement freestall dairies with cows fed a ration balanced to meet nutritional requirements and milked 2 times (MO) or 3 times (KS) daily. Milk production was measured using electronic meters in the milking parlor on both farms. Cases of metritis were identified by the herdsman based on clinical signs (fetid red-brown watery vaginal discharge with a flaccid uterus). The diagnosis was confirmed by a trained veterinarian from the University of Missouri who performed rectal palpation and examined the uterus and uterine discharge of the selected cows. The same individual was responsible for diagnosing metritis on both dairies. Cows with a clinical diagnosis of metritis between 5 and 10 d postpartum (n = 18; fetid red-brown watery vaginal discharge with a flaccid uterus; adapted from Kelton et al., 1998) were enrolled in the study. The metritis group included 11 cows with a diagnosis of retained placenta (failure to pass the placenta out of the uterine lumen within 24 h after calving). Cows with metritis were matched with clinically healthy postpartum cows (n = 17; viscous [not watery] and nonfetid discharge) that calved during the same week. None of the healthy cows had retained placenta. Rectal temperature was measured using a digital thermometer (Equate, Walmart, Bentonville, AR).

Cows were either treated with an antibiotic (ceftiofur hydrochloride, 2.2 mg/kg i.m. for 3 d; [healthy, n = 9, and metritis, n = 9) or were left untreated (healthy, n = 8, and metritis, n = 9; Supplemental Figure S1, see Notes) to create a 2 × 2 factorial arrangement of treatments (metritis-antibiotic, healthy-antibiotic, metritis-untreated control, and healthy-untreated control). For cows originating from eastern Kansas, diagnosis was performed within the Kansas herd, cows were moved on the same day to the University of Missouri dairy herd, and antibiotic treatment was administered at the University of Missouri. Healthy control cows were examined thrice weekly for symptoms of metritis following the initial diagnosis. One untreated healthy cow developed metritis on d 11, and this cow was included in the metritis group. A listing of specific days postpartum for assignment to the study and treatment is provided in Supplemental Figure S1. Cows remained in the University of Missouri herd until ~1 mo postpartum.

### Sample Collection at Disease Diagnosis

The cows enrolled in the study had their uterine contents sampled for 16S rRNA gene sequencing when they were identified and selected for the trial. Uterine swabs were collected transcervically using a double-guarded culture swab (Jorgensen Laboratories, Loveland, CO) following perineal cleaning and disinfection with povidone-iodine. Swab samples were carefully moved to a sterile cryovial (CryoTube Vial, Thermo Fisher Scientific, Waltham, MA) and immediately placed in dry ice and subsequently stored in a −80°C freezer.

### Blood Sample Collection and Analysis from Disease Diagnosis to Slaughter

Blood samples were collected thrice weekly. Samples were collected from the coccygeal veins (10 mL Monoject EDTA, Covidien, Mansfield, MA), and plasma was separated by centrifugation (2,000 × *g* for 15 min at 4°C) and stored at −20°C. Plasma haptoglobin concentrations were measured with a commercial ELISA kit (catalog number E-10HTP, Immunology Consultants Laboratory Inc., Portland, OR) following the manufacturer’s protocol. Plasma progesterone concentrations were measured using a validated radioimmunoassay (Pohler et al., 2013). The intra- and interassay CV were 5.5 % and 12.0% for progesterone and 3.4% and 1.5% for haptoglobin.

### Sample Collection After Slaughter

Cows were humanely slaughtered at the University of Missouri abattoir by captive bolt stunning and exsanguination at 29.1 ± 1.7 d postpartum. The uterus was removed from the abdomen by transecting the vagina ~10 cm from the cervical os. The entire tract was wrapped in a surgical drape, placed on ice, and carried to the microbiology laboratory of the Veterinary Medical Diagnostic Laboratory at the College of Veterinary Medicine at the University of Missouri (Columbia, MO; located ~250 m from the abattoir). The uterus and ovaries were placed inside a biosafety cabinet, and the surgical drape was unwrapped to expose the uterine surface. The external surface of the uterus (**EXT**) was sampled for bacteriological culture using a sterile culture swab. Afterward, a tissue sample from the EXT surface near the tip of the horn was collected using sterile forceps and a scalpel, inserted into a sterile CryoTube, and frozen in liquid nitrogen to be used for 16S rRNA DNA sequencing. The outside of the uterus was then cleaned and disinfected with povidone-iodine, and the uterine lumen was exposed by dissecting through the midsection of the horn (near the curvature) and into the uterine lumen with a scalpel. A sample of the uterine lumen was collected using sterile forceps to hold the tissue and a scalpel to cut the tissue sample from the endometrium. Both the previously gravid and nongravid uterine horns were collected for bacteriological culture, and a second sample was collected, placed in a CryoTube, and frozen in liquid nitrogen for 16S rRNA DNA sequencing. Duplicate samples for 16S rRNA DNA sequencing were collected, and all samples were immediately snap frozen in liquid nitrogen and then transferred to a −80°C freezer for storage. The initial collection in the microbiology laboratory required ~30 min.

Following the collection of samples for bacteriology and 16S rRNA gene sequencing, the reproductive tract was transported to a second laboratory in the Animal Science Research Center of the University of Missouri (~500 m from the microbiology laboratory; Columbia, MO) where the uterine lumen was flushed with 10 mL of cell culture–grade PBS (Gibco, ThermoFisher Scientific). The flush was recovered into a Petri dish and classified visually as either clear (nonpurulent, no pus) or containing purulent material (pus). Furthermore, cows with uterine flush resembling acute metritis (fetid, reddish-brownish discharge) were defined as “acute infection.” A longitudinal incision was made along each horn, and the lumen of the uterus was inspected visually to confirm the original flush phenotype. Samples of endometrium were then collected from the gravid and nongravid horns and fixed in 10% neutral buffered formalin. Sites for collection were at the midpoint of the horn. The additional collections in the Animal Science Laboratory required ~1 h. The fixed tissues were trimmed to ~2 cm of endometrial surface, including caruncular and intercaruncular regions. The tissue samples were then placed into a plastic tissue cassette and taken to the Veterinary Medicine Diagnostic Laboratory at the University of Missouri (Columbia, MO) for paraffin embedding and hematoxylin and eosin staining. A board-certified theriogenologist (T. J. Evans; working independently and blinded to the health status, treatment, or uterine flush phenotype) scored the amount of inflammation in the uterine epithelium and stroma. Endometrial inflammation (total number of eosinophils, neutrophils, lymphocytes, and plasma cells counted in ten 400× fields from a section of gravid and nongravid horn) was scored. The inflammation score was 0.5, 1, 1.5, 2, 2.5, and 3 for cows with an average of <10, 10 to 19.9, 20 to 29.9, 30 to 39.9, 40 to 49.9, and >50 cells per field, respectively. Endometrial fibrosis was also scored as rare (score = 1), rare to occasional (1.5), occasional (score = 2), occasional to frequent (2.5), or frequent (3). Fibrosis for cows scored as 1, 1.5, or 2 always had <5 layers of fibroblasts. Fibrosis for cows scoring 2.5 or 3 generally had from 5 to 10 layers of fibroblasts. The scoring system was adapted from the original scoring system of R. M. Kenney (Snider et al., 2011).

### Bacterial Culture

Tissue samples were ground in a sterile tissue grinder with brain-heart infusion (**BHI**) broth and inoculated into both solid media and broth for incubation. Swab samples were handled identically except for tissue grinding, and the swabs themselves were incubated in thioglycolate broth. All samples were plated onto tryptic soy agar with 5% sheep blood (**TSA**), MacConkey agar, phenylethyl alcohol agar (**PEA**), and thioglycolate broth for incubation under aerobic conditions. Aerobic cultures were incubated at 36°C in a standard ambient air incubator. Capnophilic cultures were maintained at 36°C under 5% CO_2_. *Campylobacter* cultures were placed in Mitsubishi boxes equipped with a microaerophilic sachet (Mitsubishi AnaeroPak MicroAero gas generator, Remel, Lenexa, KS), providing 6% to 12% O_2_ and 5% to 8% CO_2_, and then incubated at 42°C for enteric *Campylobacter* and 35°C for reproductive *Campylobacter*. Samples were also plated onto TSA and PEA for incubation under anaerobic conditions; chocolate agar, Hayflick agar, and BHI broth for incubation under 5% CO_2_; and selective *Campylobacter* agar for incubation under microaerophilic conditions. Anaerobic cultures were held in Mitsubishi boxes using a Mitsubishi AnaeroPack anaerobic gas generator (Remel, Lenexa, KS), and anaerobic conditions (<1% oxygen, >15% CO_2_) were verified using anaerobic indicators (Remel, Lenexa, KS). All bacterial culture media used were sourced from Remel (Lenexa, KS), except for the reproductive *Campylobacter* medium, which was obtained pre-reduced from Anaerobe Systems (Morgan Hill, CA). Media were incubated for 7 d and evaluated daily. All isolates were identified via MALDI-TOF mass spectrometry, standard biochemical tests, or 16S rRNA sequencing. The number of colonies present for each isolate were counted if there were fewer than 50 colonies per plate and estimated by using a transilluminated grid colony counter if more than 50 colonies per plate. If more than 50, nonadjacent squares on the grid counter were counted and the number of colonies was averaged and multiplied by 30 (total number of squares on the plate).

### 16S rRNA Gene Sequencing

A manual precipitation protocol was used for DNA extraction (Yu and Morrison, 2004). Library construction and sequencing were performed by the University of Missouri DNA Core. A Qubit double-stranded DNA broad range assay (Life Technologies, Carlsbad, CA) was used to determine DNA concentration. Samples were normalized to 3.51 ng/μL DNA for PCR amplification. The V4 hypervariable region of the 16S rRNA gene was amplified using single-indexed universal primers (U515F [5′-GTGCCAGCMGCCGCGGTAA-3′]; 806R [5′-GGACTACHVGGGTWTCTAAT-3′]) with standard adapter sequences (Illumina Inc., San Diego, CA). The PCR program for amplification was as follows: 98 °C (3 min) + [98 °C (15 s) + 50 °C (30 s) + 72 °C (30 s)] × 40 cycles + 72 °C (7 min). The V4 region of the 16S rRNA gene was selected for library generation because this region yields optimal community clustering (Caporaso et al., 2011). The Illumina MiSeq platform (V2 chemistry with 2 × 250-bp paired-end reads) was used to sequence pooled amplicons.

### Analysis of 16S rRNA Gene Sequences

Amplicon sequences from the V4 hypervariable region of the 16S rRNA gene were processed and analyzed using QIIME2 (version 2020.6; https://qiime2.org; Bolyen et al., 2019). Fastq files containing forward and reverse sequences were imported into QIIME2 and demultiplexed to assign sequences to samples. The plugin cutadapt (Martin, 2011) was used to trim off PCR primers (515F/806R) from raw sequences. The QIIME2 Divisive Amplicon Denoising Algorithm (DADA2) plugin was used for detecting and correcting Illumina amplicon sequencing errors (Callahan et al., 2016). The QIIME2 quality control plugin was used to exclude contaminant sequences such as host sequences (e.g., cow DNA) and nontargeted (e.g., nonbacterial) sequences. Green Genes (https://greengenes.secondgenome.com) operational taxonomic unit reference sequences (99% sequence identity; version 13_8) were used for quality control. Sequences filtered out during this step were investigated using the National Center for Biotechnology Information (**NCBI**) BLAST nucleotide database (https://blast.ncbi.nlm.nih.gov/Blast.cgi) to ensure that only contaminant sequences were removed. A total of 123 contaminant sequences were eliminated in this process (Supplemental Dataset S1, see Notes). Sequences filtered out during this step were investigated using the NCBI BLAST nucleotide database (Supplemental Dataset S2, see Notes). Predicted matching eliminated sequences included regions in bovine, fungal, and viral genomes, among others.

To perform phylogenetic diversity analyses, a rooted phylogenetic tree was generated using the QIIME2 phylogeny function after samples were rarefied to depths of 15,000 and 1,000 sequences for the experiments performed during the first week postpartum (e.g., uterine swab sample) or first month (low biomass environment) postpartum (samples collected at animal slaughter), respectively. For the swab samples collected at the time of disease diagnoses, pairwise comparisons for α-diversity measures (Pielou’s evenness, Pielou, 1966; and Faith’s phylogenetic diversity, Faith, 1992) were computed using the Kruskal–Wallis test. The unweighted UniFrac distances, a measure of β-diversity (Lozupone and Knight, 2005; Lozupone et al., 2007), were also calculated, and permutational multivariate ANOVA (**PERMANOVA**) was used in pairwise comparisons to evaluate β-diversity group distances. For the uterine samples collected at 1 mo postpartum, α- and β-diversity metrics were analyzed using methods that accounted for the paired sampling design (gravid and nongravid horn) without assuming independence between horns. The α-diversity metrics (Faith’s phylogenetic diversity and Pielou’s evenness) were evaluated using linear mixed-effects models in QIIME 2 (version 2024.10) with the “longitudinal linear-mixed-effects” plugin. The models included random intercepts for cow ID to account for within-cow correlation, fixed effects for original disease classification (healthy vs. metritis), antibiotic treatment (treated vs. untreated), uterine health status (noninfected [clear] vs. infected [purulent or acute]), their interactions, and sampling site (gravid vs. nongravid horn), using the formula “metric ~Class × TRT × Status + Site.” Models were run with “–p-state-column Site” and “–p-individual-id-column Cow_ID”, and visualizations were generated for model diagnostics. Beta diversity (unweighted UniFrac distances) was analyzed using PERMANOVA in QIIME 2’s “diversity adonis” plugin, incorporating original disease classification, antibiotic treatment, uterine health status, their interactions, cow ID, and sample site as a covariate in the formula “Class * TRT * Status + Site + Cow_ID” to control for pairing, with 999 permutations. To further quantify within-group variability, the median, first quartile, third quartile, and interquartile range (**IQR**) of the within- and between-group UniFrac distances were calculated in Python (version 3.10) for interpretation. Furthermore, principal coordinate analyses (**PCoA**) plots for qualitative (presence or absence; Jaccard distances, Jaccard, 1912) and quantitative (abundance and presence or absence; Bray–Curtis, Bray and Curtis, 1957) metrics were generated using Emperor (Vázquez-Baeza et al., 2013, 2017) to aid in data visualization and interpretation.

A preformatted taxonomy classifier (Bokulich et al., 2020) was used for assigning taxonomy classification to the 16S rRNA amplicon sequences (Pruesse et al., 2007; Pedregosa et al., 2011; Quast et al., 2013; Bokulich et al., 2018), and an amplicon sequence variant (**ASV**) table was generated (McDonald et al., 2012). Amplicon sequence variants sharing the same taxa were collapsed together (at the species level) using the QIIME2 taxa collapse function.

Differential abundance analysis on the identified ASV was performed using the analysis of composition of microbes (**ANCOM**) statistical framework (Mandal et al., 2015). For ANCOM, data were preprocessed to remove features with low reads (fewer than 10 reads across all samples), rarely observed (present in fewer than 2 samples), and with low variance (less than 10e-4). Because ANCOM is based on log ratios, the QIIME2 add-pseudocount plugin was used to add one count to every feature, allowing ANCOM analysis to be performed on features with zero counts. Pairwise comparisons using the ANCOM framework were conducted to compare the microbiome of samples from different health statuses (e.g., metritis versus healthy at clinical diagnosis; and clear versus purulent flush at 1 mo postpartum). The QIIME2 EMPress plugin (Cantrell et al., 2021) was used to generate a phylogenetic tree for exploring the hierarchical evolutionary relationships of features subjected to ANCOM.

A supervised machine learning approach using multinomial regression analysis was conducted using Songbird (Morton et al., 2019) to investigate differences in microbial composition within the uterine lumen. The analytical approach implemented by Songbird (Morton et al., 2019) was based on ranking microbes relative to each other within a sample. Relative differentials (logarithms of the fold change in abundance of taxa between 2 conditions) were estimated using a multinomial regression, and the coefficients from the multinomial regression were ranked to determine which taxa changed the most within samples and across conditions. Three distinct models were generated using the standalone version of Songbird. The first model, termed the “null model,” was created to represent random chance (this model was run without any metadata). The null model was used for comparison to evaluate whether additional models containing selected variables presumed to be associated with uterine disease at 1 mo postpartum were more predictive than random chance. The second model contained only the categorical information of flush phenotype, as a clear uterine fluid is an evident sign of a healthy uterus, a flush containing purulent material is indicative of infection, and a flush with fluid resembling early postpartum metritis (acute disease) is strong evidence of severe infection. Lastly, a third model, termed the “full model,” was created containing variables representing the flush phenotype (clear, purulent, or acute), cow identification, disease status 1 wk postpartum (healthy versus metritis), antibiotic therapy (treated versus not treated), and plasma progesterone concentrations at the time of slaughter (indicative of cyclicity; based on analyses of thrice-weekly plasma progesterone analyses). Outputs from the 3 models were evaluated using TensorBoard, a visualization toolkit of Tensorflow (Abadi et al., 2016), an open-source machine learning library developed by the Google Brain team (Mountain View, CA). TensorBoard provided measurements and visualizations needed for a machine learning workflow, including metrics such as loss and accuracy that were carefully evaluated for each model tested. Furthermore, the interactive web application Qurro (Fedarko et al., 2020) was used to plot the differentials generated in Songbird.

Songbird was also run as a QIIME2 plugin, and the pseudo-Q^2^ score was calculated for the 2 models tested against the null model. The pseudo-Q^2^ score indicates the predictive accuracy of the model. It is calculated using the formula Q^2^ = 1 − m1/m2, where m1 indicates the average absolute error for the testing model and m2 indicates the average absolute error for the null model (representing random chance). A pseudo-Q^2^ score close to 1 indicates high predictive accuracy of the testing model, whereas a pseudo-Q^2^ score that is low or below zero indicates poor predictive accuracy and possible overfitting.

### Additional Statistical Analyses

The sum of 16S sequencing reads identified per cow was analyzed using a mixed-effects model implemented in PROC MIXED (SAS 9.4, SAS Institute, Cary, NC). The model included fixed effects for original disease diagnosis (healthy vs. metritis), antibiotic treatment (treated vs. not treated), uterine flush (clear vs. purulent + acute), location (gravid vs. nongravid horn), and their interactions (up to the 3-way), with degrees of freedom adjusted using the Kenward–Roger method to account for small sample sizes and potential imbalances. Because 2 samples were collected from each cow, the random effect of cow was incorporated to address the nonindependence of observations within individual animals. Least squares means for original disease diagnosis, antibiotic treatment, flush phenotype, location, and their interactions were estimated, with pairwise comparisons adjusted using Tukey’s method. Additionally, predicted values of the sum of reads were generated for each observation and summarized by cow using PROC MEANS to obtain the estimated sum of reads inside the uterine lumen for each cow, providing cow-level estimates adjusted for the fixed and random effects in the model.

The data for the inflammation and fibrosis scoring of the endometrium were categorical and therefore analyzed using PROC GENMOD of SAS with a statistical model that included original disease diagnosis, flush phenotype, antibiotic treatment, treatment by disease, treatment by flush, and location. Cow nested within status by treatment by flush was included as a repeated variable. The compound symmetry covariance structure was used.

The prevalence of bacterial species found in at least 3 cows (bacterial culture data) was tested for the effect of initial disease diagnosis (healthy vs. metritis), treatment (antibiotic vs. control), and their interaction using PROC MIXED. The same data were tested for flush phenotype (clear vs. purulent + acute), treatment (antibiotic vs, control), and interaction (PROC MIXED).

The total number of bacterial species isolated from each cow and the total number of colonies from each cow were analyzed for the effects of the initial disease diagnosis, flush phenotype, and treatment. Culture data from the previous gravid and nongravid uterine horns were combined for the analysis. Samples from the external surface of the uterus were used as a control for comparison. The bacteriology data were not normally distributed (positive skew; results of the “normal” command within PROC UNIVARIATE of SAS), so data were log_10_ transformed before analysis to achieve a skewness and kurtosis from −1 to 1. Data were subsequently analyzed using PROC MIXED of SAS for the effects of original disease diagnosis, flush phenotype, antibiotic treatment, treatment by disease, and treatment by flush. In all analyses, significance was defined as *P* ≤ 0.05 and tendencies as 0.05 < *P* < 0.10.

### Post Hoc Power Analyses

A series of post hoc power analyses on the 16S rRNA sequencing (Kelly et al., 2015), inflammation scoring, and bacteriology were conducted to assess whether the study was adequately powered to address the primary research questions. A detailed description of all power analyses performed is provided in Supplemental Appendix S1 (see Notes).

## RESULTS

### Rectal Temperature, Haptoglobin, Milk Yield, and Progesterone

Rectal temperatures (mean ± SD) at the time of disease diagnosis were 38.7°C ± 0.7°C for cows with metritis and 38.6°C ± 0.4°C for healthy cows. Plasma concentrations of haptoglobin (mean ± SD) at the time of diagnosis were 2.5 ± 1.4 g/L for metritis cows and 0.2 ± 0.4 g/L for healthy cows. The average milk production (mean ± SD) for the first month postpartum was 21.6 ± 6.6 kg/d for metritis cows and 24.5 ± 6.6 kg/d for healthy cows. Based on plasma progesterone, 3 out of 17 metritis cows and 7 out of 18 healthy cows were cycling (>1 ng/mL progesterone) at the time of slaughter.

### Uterine Microbiota at Disease Diagnosis

**Figure 1.**
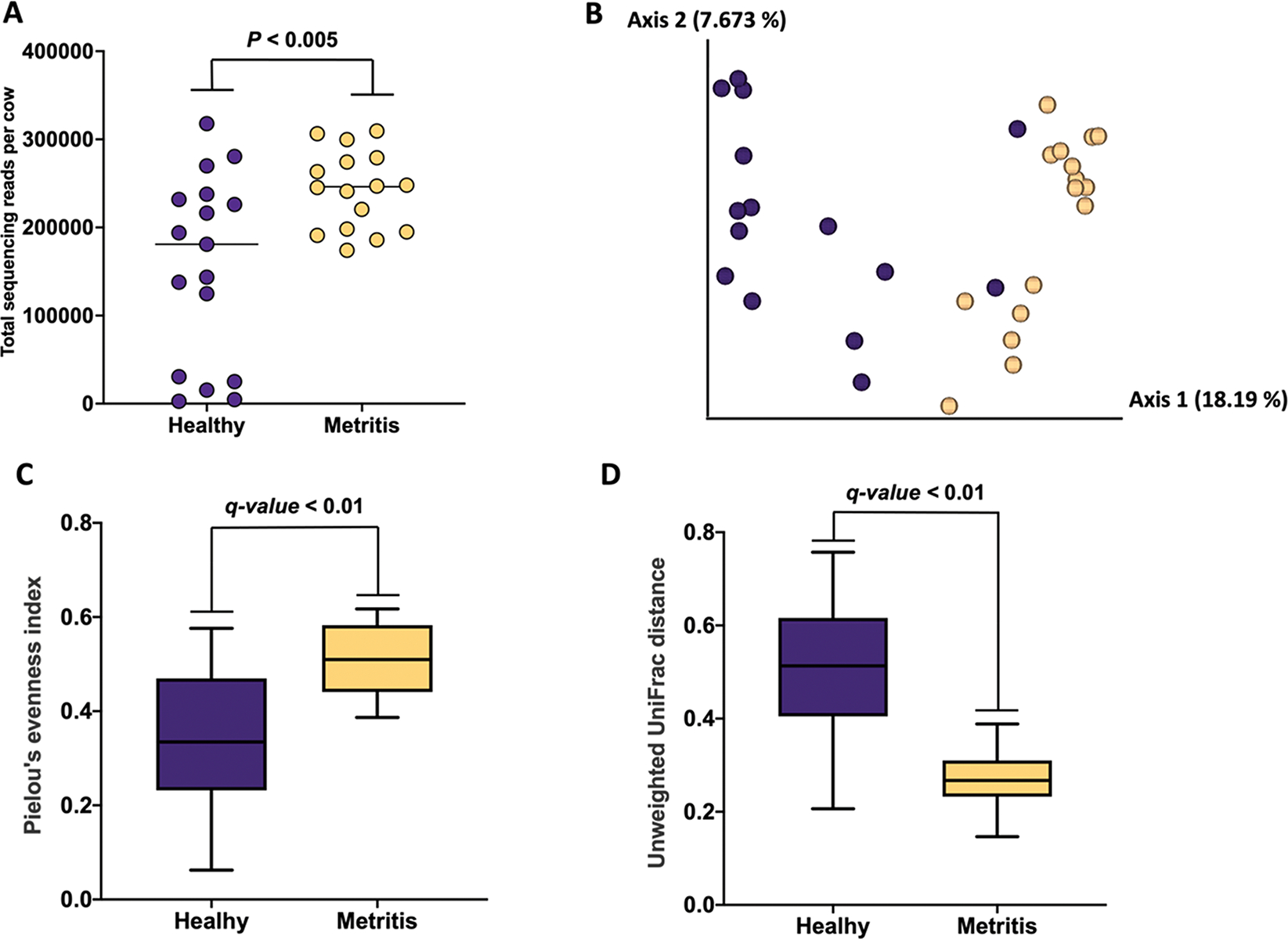
Sum of sequencing reads per cow according to disease status (healthy vs. metritis; A), Jaccard principal coordinate analysis (PCoA) plot (B), Pielou’s index (C), and unweighted Unifrac distances (D) from uterine swabs collected from metritis (blue symbols and bars) and healthy (yellow symbols and bars) cows at the time of disease diagnosis. Of note, swab samples were not collected for 2 cows diagnosed with metritis. Horizontal lines in panel A indicate the median. Box and whisker plots were generated using GraphPad Prism, where the box represents the interquartile range (IQR, spanning the 25th to 75th percentiles), the midline within each box denotes the median, and the whiskers extend to the minimum and maximum values within 1.5 times the IQR from the quartiles.

A total of 541 ASV were detected in uterine swab samples collected at the time cows were examined for metritis (Supplemental Dataset S3, see Notes). Cows diagnosed with metritis had a greater overall number of sequencing reads (*P* < 0.005) when compared with healthy cows (QIIME2). The Jaccard and Bray–Curtis PCoA plots demonstrated differences in microbial composition between metritis and healthy cows, as evidenced by the clustering of individual cows from the respective groups (Figure 1B and Supplemental Figure S2 [see Notes], respectively). Figures 1A and 1B display 16 metritis samples instead of the reported 18, as one cow was excluded from the d-7 analysis due to a delayed metritis diagnosis (started exhibiting clinical signs on d 11) and one cow lacked a d-7 uterine swab for sequencing. Furthermore, in Figure 1B, 2 samples of healthy cows were excluded during the rarefaction step of the diversity analyses, as they had fewer than 15,000 reads.

The Pielou’s index was greater in samples of cows with metritis compared with healthy cows (q-value <0.01; QIIME2), indicating that the abundance of ASV detected in cows with metritis was more even compared with ASV detected in healthy cows. In contrast, the unweighted UniFrac distance was greater (q-value <0.01) in healthy compared with metritis cows (Figure 1D). This result indicates that the microbiome of metritis cows was less diverse than the microbiome of healthy cows.

**Figure 2.**
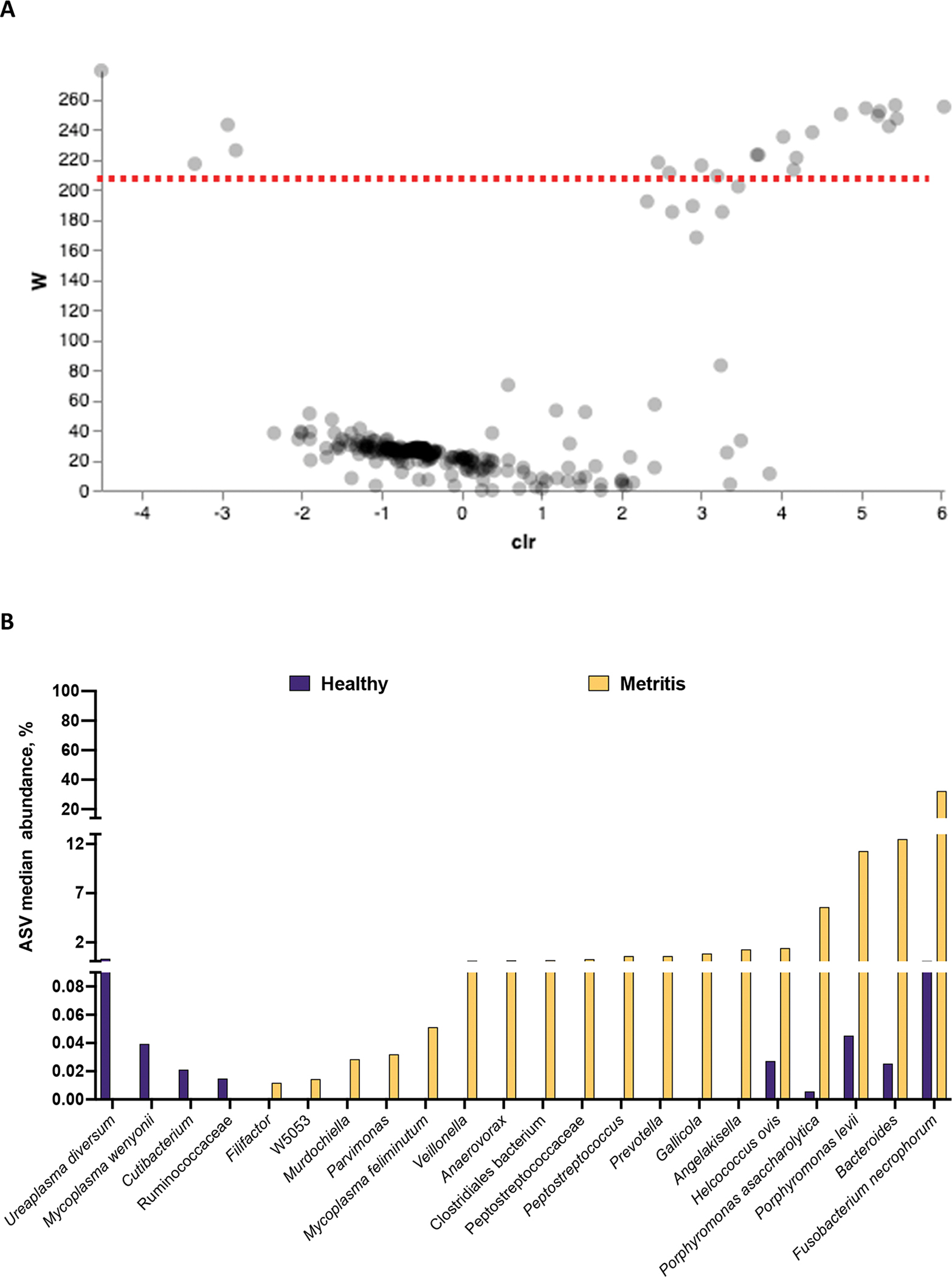
Differential abundance analysis with ANCOM for microbiomes collected from uterine swabs performed at the time of disease diagnosis. (A) Volcano plot highlighting the 22 differently abundant ASV (18 increased and 4 decreased in metritis compared with healthy cows). The red dotted line represents the cutoff for the W-value (y-axis) indicating statistical significance (approximating *P* ≤ 0.05 in traditional tests; features above the dotted line were significantly different between metritis and healthy cows). The W-value represents a count for the number of times that the null hypothesis was rejected for a given ASV. The null hypothesis states that the average abundance of a given ASV is equal in metritis and healthy cows. The x-axis displays the centered log ratio (clr) of the abundance of a given ASV between healthy and metritis cows. A negative clr means an ASV (each gray dot in the volcano plot) is more abundant in healthy than metritis cows, and a positive clr means that the ASV is more abundant in the metritis compared with healthy cows. There were 6 ASV that approached significance tending to increase in metritis compared with healthy cows (*Filifactor*, *Peptococcus simiae*, SR1 bacterium, *Clostridium cadaveris*, *Tissierella*, and *Helcococcus*). (B) Histogram plotting the median abundance of ASV that were significantly different between healthy and metritis cows based on ANCOM. The displayed abundances (y-axis) were calculated as the median for the percentage of reads mapped to each ASV of interest out of the total reads sequenced per cow.

Results from the differential abundance analysis using ANCOM are summarized in Figures 2A and 2B. There were 22 differentially abundant ASV identified by ANCOM (Figure 2A). From the 22 differently abundant features, 18 were increased in the uterus of cows with metritis (Figure 2B). An additional 6 ASV approached significance (W-values ranging from 168 to 202) and tended to be increased in metritis compared with healthy cows (*Filifactor*, *Peptococcus simiae*, *SR1 bacterium*, *Clostridium cadaveris*, *Tissierella*, and *Helcococcus*; Supplemental Table S1, see Notes). The dysbiosis associated with metritis was marked by a notable change in the microbial community for only a small population of bacteria (~28 out of 541 detected ASV).

**Figure 3.**
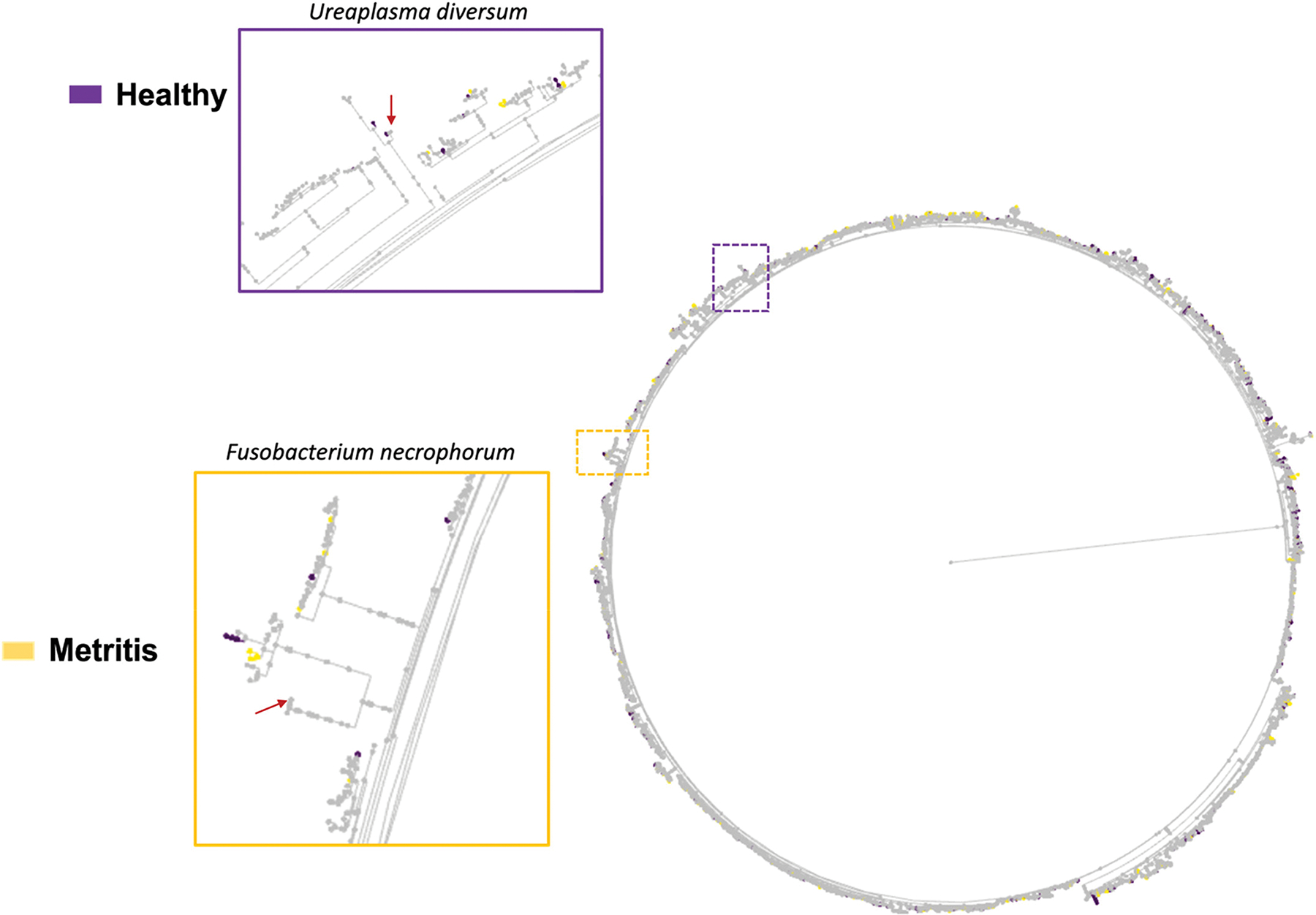
A phylogenetic tree showing the hierarchical evolutionary relationships for the features subjected to ANCOM. The outermost tips of the tree are termed “leaves” and represent a unique ASV. The purple and yellow dotted squares represent areas of the phylogenetic tree shown in the insets. The red arrows point to the leaves representing the *Ureaplasma diversum* (purple square) and *Fusobacterium necrophorum* (yellow square), the most differently abundant ASV that increased in healthy and metritis cows, respectively. Importantly, gray leaves represent ASV that were identified in both healthy and metritis cows, whereas leaves that are purple or yellow represent ASV that were uniquely observed in either healthy or metritis cows, respectively.

The phylogenetic tree, generated using EMPress (Cantrell et al., 2021), is presented in Figure 3. Gray leaves represent ASV that were identified in both healthy and metritis cows, whereas leaves colored purple or yellow represent ASV that were uniquely observed in either healthy or metritis cows, respectively. The majority of the ASV identified were present in both healthy and metritis cows (most of the leaves in the phylogenetic tree were gray) with few purple and yellow leaves spread out across the tree.

### Morphological Observations and Histological Analyses of Uterine Tissue (1 mo Postpartum)

There were 20 (57%), 12 (34%), and 3 (9%) cows that were defined as having a clear, purulent, or acutely infected uterine lumen at tissue collection, respectively (Table 1). There were 5 healthy cows (29%) with a purulent flush and 10 metritis cows (56%) with a purulent or acutely infected uterus at the time of tissue collection. The number of cows in the clear, purulent, or acute infection groups was identical or nearly identical for antibiotic-treated and untreated cows, but the study was underpowered to measure the effect of antibiotic treatment (Table 1). We found no effect of the original disease diagnosis (healthy vs. metritis) on the uterine histological score for fibrosis, epithelial inflammation, stromal inflammation, or caruncular inflammation (Table 2). The eosinophil score was greater (*P* < 0.006) for cows with metritis than healthy cows. Fibrosis score (*P* < 0.020), epithelial inflammation score (*P* < 0.001), stromal inflammation score (*P* < 0.022), caruncular inflammation score (*P* < 0.034) and eosinophil score (*P* < 0.001) were greater for cows with a purulent or acutely infected flush compared with a clear flush. We detected interactions of disease diagnosis by treatment for epithelial inflammation score (*P* < 0.025), stromal inflammation score (*P* < 0.013), caruncular inflammation score (*P* < 0.017), and eosinophil score (*P* < 0.041), where antibiotic treatment increased these scores in metritis cows but decreased these scores in healthy cows (Table 2). We observed a greater fibrosis score (2.61 ± 0.05 vs. 2.44 ± 0.07; *P* < 0.027) and a greater eosinophil score (1.46 ± 0.07 vs. 1.23 ± 0.07; *P* < 0.001) in the gravid horn (gravid vs. nongravid; effect of location).

### Bacterial Culture of Tissue at Slaughter (1 mo Postpartum)

A complete list of cultured bacteria from all locations (external surface, gravid horn, and nongravid horn) is provided (Supplemental Table S2, see Notes). Many bacteria were isolated from the external surface of the reproductive tract (contamination that arose during tissue collection; Supplemental Table S3, see Notes). We found no effect (*P* > 0.10) of the original disease diagnosis or the antibiotic treatment on the average prevalence of the major species found on the external surface of the tract. Within the uterine lumen, no growth was found for 8 of 17 (47%) healthy cows and 3 of 18 (17%) metritis cows. There were fewer (*P* < 0.01) 16S rRNA gene sequence reads in cows that had no growth for any bacteria (n = 11; 42,426 ± 56,363) compared with cows that had growth of any bacteria (n = 24; 229,656 ± 36,158) in uterine tissue samples. For the most-prevalent species (Table 3), cows with early postpartum metritis had a greater prevalence (21.3% ± 2.5%) compared with healthy cows (2.7% ± 2.5%; *P* < 0.001), and there was no effect (*P* = 0.148) of antibiotic treatment or antibiotic by group (*P* = 0.174) on the average prevalence of the listed species (Table 3).

Forty-five percent of cows with a clear uterine flush had no growth on bacterial culture, compared with 13% for cows with a purulent lumen or an acute infection (Table 4). For the most prevalent species (Table 4), cows with a purulent or acute uterus had a greater prevalence (24.1% ± 2.8%) compared with clear lumen cows (5.4% ± 2.8%; *P* < 0.001). We found an antibiotic by group interaction (*P* = 0.0312) on the average prevalence of the listed species because antibiotic treatment had no effect on the prevalence of species in clear flush cows (5.4% ± 3.9% vs. 5.4% ± 3.9%) but antibiotic treatment decreased the prevalence in purulent or acute cows (15.4% ± 3.9% vs. 32.9% ± 3.9%; antibiotic vs. untreated, respectively).

An analysis was carried out that included all cultured species and a cumulative count of all colonies observed. As expected, when data for the external surface of the tract (contamination control) were analyzed, no effect of initial disease diagnosis (healthy or metritis) or flush phenotype (clear or purulent + acute) was found on the number of species isolated or the cumulative count of colonies from individual cows (Supplemental Table S4, see Notes). In contrast, there was an effect of the initial disease diagnosis on both the number of species isolated and the total colony count in uterine lumen (*P* < 0.0001; Table 5). The flush phenotype “clear” was associated with the isolation of fewer species of bacteria (*P* < 0.0302), and a tendency for fewer colonies (*P* < 0.0886) was observed. We observed a status by treatment interaction (*P* < 0.0306), because the number of colonies was decreased in cows with metritis treated with antibiotics, but antibiotic treatment had no effect on the number of colonies counted for healthy cows (Table 5).

### Uterine Microbiota of the External Surface of the Tract and Uterine Lumen (1 mo Postpartum)

A total of 1,560 ASV detected in the uterine horns and external control samples following tissue collection 1 mo postpartum (Supplemental Dataset S4, see Notes). The Jaccard (Supplemental Figure S3A, see Notes) and Bray–Curtis (Supplemental Figure S3B) PCoA plots demonstrated similarities in microbial composition between samples from the previously gravid (light blue symbols) and nongravid (light green symbols) horns, and differences in the microbial composition of samples from the uterine horns compared with external control samples (gray symbols). Pielou’s evenness (Supplemental Figure S3C) was increased in control external samples compared with samples collected from the lumens of the gravid and nongravid horns (q-value <0.01), but was not different between the gravid and nongravid horns (q-value = 0.63). Similarly, Faith’s phylogenetic diversity (Supplemental Figure S3D; Faith, 1992) was also increased in the external control samples compared with samples collected from the gravid and nongravid horns (q-value <0.01), but was not different between the gravid and nongravid horns (q-value = 0.63). The unweighted UniFrac distances (Supplemental Figure S3E) among external control samples were also increased compared with samples from the lumens of the gravid and nongravid horns (q-value <0.01), but not different between the gravid and nongravid horns (q-value = 0.98).

We found no differences in microbial composition between the previously gravid and nongravid uterine horns (results from the differential abundance analyses with ANCOM). There were 12 ASV significantly increased in external control compared with the previously gravid horn samples (Supplemental Table S5, see Notes), and 11 ASV increased and 1 ASV decreased in the external control samples compared with the nongravid horn (Supplemental Table S6, see Notes). Most of the ASV (external vs. gravid or nongravid horn) were identical.

### Effect of Original Disease Diagnosis, Antibiotic Treatment, and Flush Phenotype on Uterine Microbiota at Tissue Collection (1 mo Postpartum)

**Figure 4.**
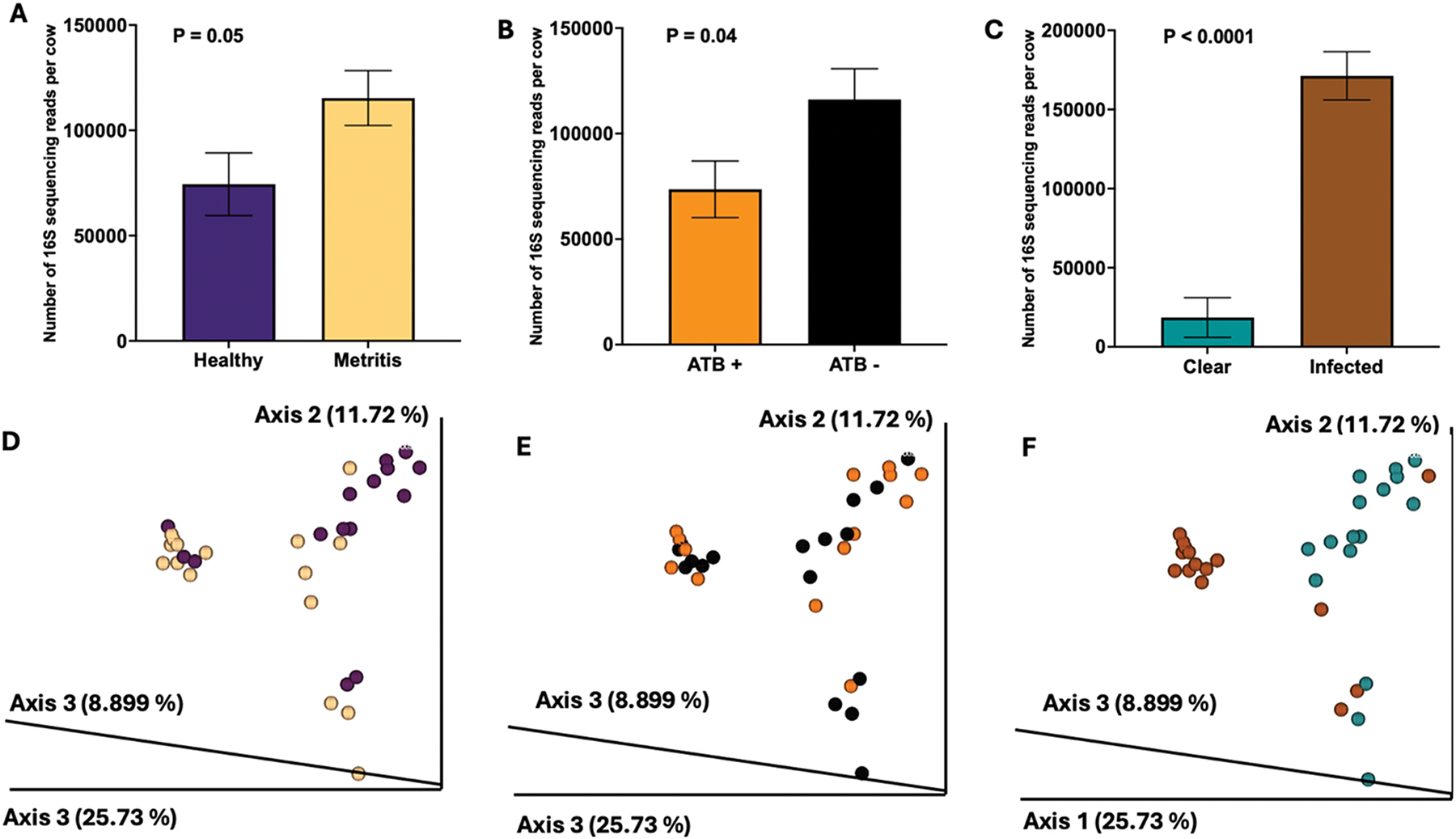
Uterine microbiota at 1 mo postpartum in cows diagnosed at 5 to 10 d postpartum as healthy or with metritis, with or without antimicrobial treatment at time of disease diagnosis. Estimated 16S rRNA sequencing read counts are shown by (A) original disease classification (healthy vs. metritis), (B) antimicrobial treatment (treated [ATB+] vs. not treated [ATB−]), and (C) uterine discharge appearance: clear fluid or infected (pus, or reddish fetid discharge resembling metritis). Bray–Curtis principal coordinate analysis plot for the original disease classification (D), antimicrobial treatment (E), and uterine discharge appearance (F). The symbol colors in D, E, and F correspond to the bar colors in A, B, and C. Error bars indicate SE.

Regarding the microbiome of samples collected 1 mo postpartum, cows that had been diagnosed with metritis had a greater number of 16S sequencing reads in uterine samples compared with healthy cows (Figure 4A; *P* < 0.05). We also found an effect of antibiotic treatment (Figure 4B; *P* < 0.04), with untreated cows having a greater number of reads compared with treated. The uterine flush content had a large effect (Figure 4C; *P* < 0.0001) on the number of 16S sequencing reads. Although no interaction was observed between original disease diagnosis and antibiotic treatment (*P* = 0.22) or between antibiotic treatment and flush phenotype (*P* = 0.26), a significant 3-way interaction was observed among original disease diagnosis, antibiotic treatment, and flush phenotype (*P* < 0.018). Specifically, among purulent + acute cows treated with antibiotics at the time of disease diagnosis, those classified with metritis had more reads than those that had been classified as healthy (*P* < 0.0015; adjusted *P* < 0.0278 by Tukey–Kramer). In contrast, among cows not receiving antibiotic treatment but later found to be infected (purulent + acute) 1 mo postpartum, there were no differences in the number of reads between metritis and healthy cows.

Alpha diversity metrics were analyzed using linear mixed-effects models with random intercepts for cow, including fixed effects for original disease diagnosis, antibiotic treatment, flush phenotype, their interactions, and sample location. This approach accounted for the paired sampling design without assuming independence between horns. No significant main effects of original disease diagnosis (*P* = 0.504), antibiotic treatment (*P* = 0.450), or flush phenotype *(P* = 0.322) were observed on Faith’s phylogenetic diversity in uterine samples collected 1 mo postpartum. However, the 3-way interaction between original disease classification, antibiotic treatment, and flush phenotype was significant (*P* < 0.020).

A similar linear mixed-effects model used for Faith’s phylogenetic diversity was applied to Pielou’s evenness, with random intercepts for cow to account for the paired sampling design without assuming independence between horns. We observed a main effect of uterine health status (clear vs. purulent + acute; *P* < 0.035), where infected cows (purulent + acute) had less evenness (coefficient = −0.337 ± 0.160) compared with noninfected (clear) cows. However, we found no effects of original disease classification (*P* = 0.91), antibiotic treatment (*P* = 0.67), their interaction (*P* = 0.32), or location (*P* = 0.53) on Pielou’s evenness in uterine samples collected 1 mo postpartum. The 2-way interactions (e.g., original disease classification by flush phenotype, *P* = 0.980); antibiotic treatment by flush phenotype, *P* = 0.881) and 3-way interaction (*P* = 0.358) were also not significant.

Regarding β-diversity, unweighted UniFrac distances were analyzed using PERMANOVA to address the paired sampling design without assuming independence between horns, with a formula including original disease diagnosis, antibiotic treatment, flush phenotype, sample location, and cow as a covariate. Significant effects of health status (pseudo-*F* = 6.72, R^2^ = 0.055, *P* < 0.001), antibiotic treatment (pseudo-*F* = 1.88, R^2^ = 0.015, *P* < 0.041), flush phenotype (pseudo-*F* = 11.88, R^2^ = 0.097, *P* < 0.001), and individual cow effects (pseudo-*F* = 2.61, R^2^ = 0.596, *P* < 0.001) were observed on microbial community structure in uterine samples collected 1 mo postpartum, explaining a total of 76.8% of the variance. Location was not significant (pseudo-*F* = 1.13, R^2^ = 0.009, *P* = 0.303), and residuals accounted for 22.8% of variance. Within-group median UniFrac distances were greater in metritis cows (median = 0.439, IQR = 0.170) compared with healthy cows (median = 0.419, IQR = 0.092), indicating greater β-diversity (i.e., more dissimilarity among microbial communities) within the metritis group. This pattern is evident in the Bray–Curtis PCoA plot (Figure 4D), where samples from cows diagnosed with metritis were more widely dispersed. Between-group distances (median = 0.447, IQR = 0.115) reflected significant dissimilarity between healthy and metritis communities overall. However, according to ANCOM, the only differentially abundant feature between healthy and metritis cows at 1 mo postpartum was from the genus *Cutibacterium*, which had increased abundance in the uterine lumen of healthy compared with metritis cows.

For antibiotic treatment, within-group median unweighted UniFrac distances were less in treated (median = 0.428, IQR = 0.111) compared with untreated (median = 0.440, IQR = 0.139) cows suggesting treatment reduces overall community variability (Figure 4E). The between-group distances (median = 0.437, IQR = 0.111) indicated differences between treatment groups but, based on ANCOM, there were no differentially abundant features in uterine samples collected at 1 mo postpartum for antibiotic-treated and untreated cows.

For flush phenotype, within-group median distances were greater in clear flush cows (median = 0.428, IQR = 0.071) compared with purulent + acute cows (median = 0.367, IQR = 0.266) indicating greater dissimilarity between noninfected (clear flush) and infected (purelent + acute) cows (Figure 4F). When the infected group was further classified according to flush appearance (pus or acute), samples from each flush phenotype category (clear, purulent, and acute) distinctly clustered in the Bray–Curtis PCoA plot (Supplemental Figure S4, see Notes).

### Analysis of the Uterine Microbiota 1 mo Postpartum Using a Supervised Machine Learning Approach

**Figure 5.**
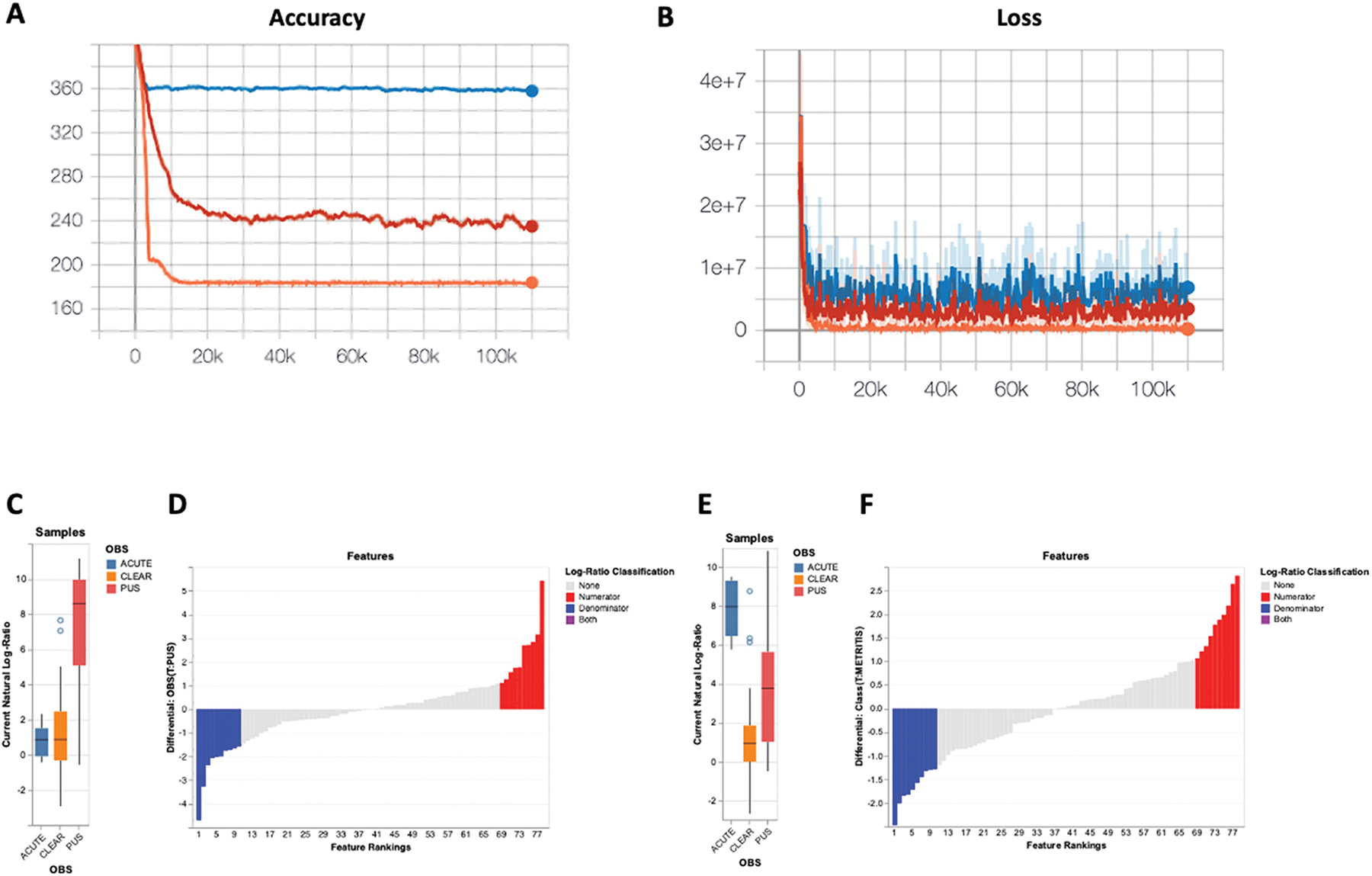
Multinomial regression analysis of the uterine microbiota at 1 mo postpartum. (A) Graph representing the prediction accuracy (capacity of the model to predict the observed read counts in samples) for the null model (blue line), a model with only the categorical information of flush phenotype (red line), and the full model (orange line). The x-axis shows the number of iterations, which represents the number of times the model trained across the entire dataset, and the y-axis shows the cross-validation values representing the accuracy of the model. (B) The loss graph represents how well each model fits the data. The y-axis is the negative log probability of the model fitting the data (a lower number indicates a better fit of the model), and the x-axis indicates the number of iterations. The sample plots (C and E) represent the log ratios (balance) of selected numerator features to denominator features, as defined by the log-ratio framework (Songbird differentials) within sample groups (clear, purulent, acute). The sample plots depict data from the top 10 ASV that were more abundant (numerator) in cows with purulent (C) and acute (E) flush phenotypes compared with cows with clear flush. The denominator features represent ASV more abundant in cows with clear flush. These plots visualize how microbial composition differs between the healthy (clear flush) and diseased (purulent and acute flush) conditions, highlighting the variation in the most differently abundant features. The rank plot (D and F), on the other hand, shows the differential abundance of selected numerator features relative to denominator features, with positive log-ratio values indicating that the numerator features (more abundant in purulent [D], Supplemental Table S6; and acute [F], Supplemental Table S7 flush phenotype) are enriched compared with the denominator features (more abundant in cows with clear flush, as shown in Supplemental Tables S6 and S7). The top 10 ASV that increased (red) or decreased (blue) are highlighted in the rank plot, with the x-axis representing the ranked features based on their differential abundance and the y-axis showing the magnitude of the log-ratio values. This provides a visual representation of the ASV that contribute most to the observed differences in microbial composition between pus versus clear (Supplemental Table S6) and acute versus clear (Supplemental Table S7) comparisons. Upper and lower edges of boxes represent the 75th percentile (third quartile, Q3) and 25th percentile (first quartile, Q1) of the log-ratio values within each categorical group, respectively. The box spans the interquartile range (IQR = Q3–Q1), encompassing the middle 50% of the data. The horizontal line inside each box denotes the median (50th percentile, Q2) of the log-ratio values for the group. Whiskers extend from the upper and lower edges of the box to the furthest data points within 1.5 times the IQR (Tukey-style). Specifically, the upper whisker reaches the maximum value ≤Q3 + 1.5 × IQR, and the lower whisker reaches the minimum value ≥Q1 – 1.5 × IQR. They illustrate the range of nonoutlier data. Dots represent outliers, log-ratio values falling outside the 1.5 × IQR range from the quartiles.

Outputs from the 3 models generated in Songbird were evaluated using TensorBoard (Figures 5A and 5B). The prediction accuracy (capacity of the model to predict the observed read counts in samples) for each model is depicted in Figure 5A. The null model, the blue line, represents random chance. The model with only the categorical information of flush phenotype (clear, purulent, or acute infection) is represented by the red line. The complete “full model” includes the information of flush phenotype, cow identification, disease status at 5 to 10 d postpartum (healthy vs. metritis), antibiotic treatment (yes or no), and plasma progesterone concentrations at the time of slaughter (indicative of cyclicity) represented by the orange line. The x-axis represents the number of iterations, which indicates the number of times the model is trained across the entire dataset. The y-axis constitutes the cross-validation values representing the accuracy of the model. For instance, the full model (orange line) is off by, on average, 170 counts, whereas the null model is off by 360 counts.

The loss function (Figure 5B) represents how well each model fits the data. The y-axis is the negative log probability of the model fitting the data (a lower number indicates a better fit of the model). The x-axis indicates the number of iterations. The full model (orange line) is closer to 0, indicating a better fit. Overall, considering the accuracy and loss graphs, the full model outperformed the null model and the model containing only the information of flush phenotype. The pseudo-Q^2^ scores generated by Songbird through QIIME2 further validate this observation: null model versus full model (Q^2^ = 0.49); null model versus flush phenotype only (Q^2^ = 0.33). A pseudo-Q^2^ score close to 1 indicates high predictive accuracy of the testing model, whereas a pseudo-Q^2^ score that is low or below zero indicates poor predictive accuracy, and possibly overfitting.

**Figure 6.**
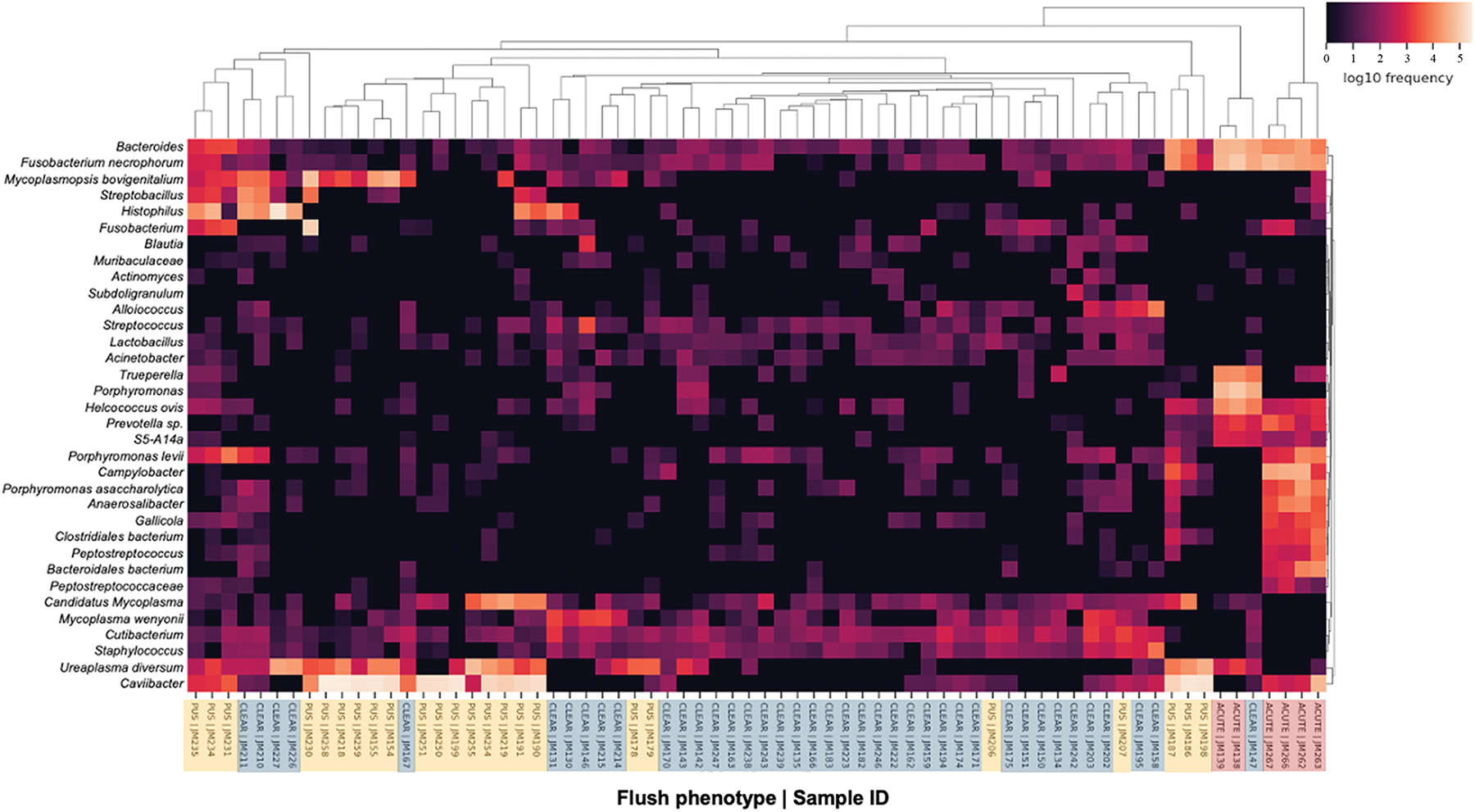
Heatmap highlighting the most differently abundant ASV among cows with clear, purulent (pus), or acutely infected flush phenotype at 1 mo postpartum based on multinomial regression (Figure 5) performed using Songbird. Samples from previous gravid (n = 35) and nongravid (n = 35) uterine horns were included. The top 10 ASV that increased or decreased in cows with purulent (pus) compared with cows with clear uterine flush phenotype, and the top 10 ASV that increased or decreased in cows with acute compared with cows with clear flush phenotype are presented in the heatmap. On the x-axis, samples are labeled by uterine flush classification: blue = clear flush, yellow = purulent flush, and red = acute flush. The Linnaean classification system was used for labeling the taxa presented in the heatmap. The species name is shown, if available. Otherwise, broader terms were used as needed for ASV identification.

Relative differentials were estimated from the full model to determine which taxa changed the most between samples and across conditions (Morton et al., 2019). The estimated log-fold change in abundance of the detected features was visualized using Qurro (Fedarko et al., 2020). The sample plots (Figures 5C and 5E) represent the log ratios (balance; based on the Songbird differentials) of selected numerator features to denominator features within sample groups (clear, purulent, acute). More specifically, these plots depict data from the top 10 ASV that were more abundant (numerator) in cows with purulent (Figure 5C) and acute (Figure 5E) flush phenotypes compared with cows with clear flush. The denominator features represent ASV more abundant in cows with a clear flush. The rank plots (Figures 5D and 5F) show the differential abundance of selected numerator features relative to denominator features, with positive log-ratio values indicating that the numerator features (more abundant in purulent Figure 5D, Supplemental Table S7 [see Notes]; and acute Figure 5F, Supplemental Table S8 [see Notes] flush phenotype) are enriched compared with the denominator features (more abundant in cows with clear flush). The top 10 ASV that increased (red) or decreased (blue) are highlighted in the rank plots, with the x-axis representing the ranked features based on their differential abundance and the y-axis showing the magnitude of the log-ratio values. This provides a visual representation of the ASV that contribute most to the observed differences in microbial composition between pus versus clear (Supplemental Table S7) and acute versus clear (Supplemental Table S8) comparisons. Of note, a total of 78 features (ASV) were ranked out of 1,560 features detected at 1 mo postpartum (x-axis Figures 5D and 5F). This was because ASV present in fewer than 10 samples, and samples with less than 1,000 reads were filtered out to improve the linear regression during the Songbird workflow. Furthermore, a heatmap for the top 30 most differently abundant features according to Songbird (Supplemental Tables S6 and S7) is presented in Figure 6.

**Figure 7.**
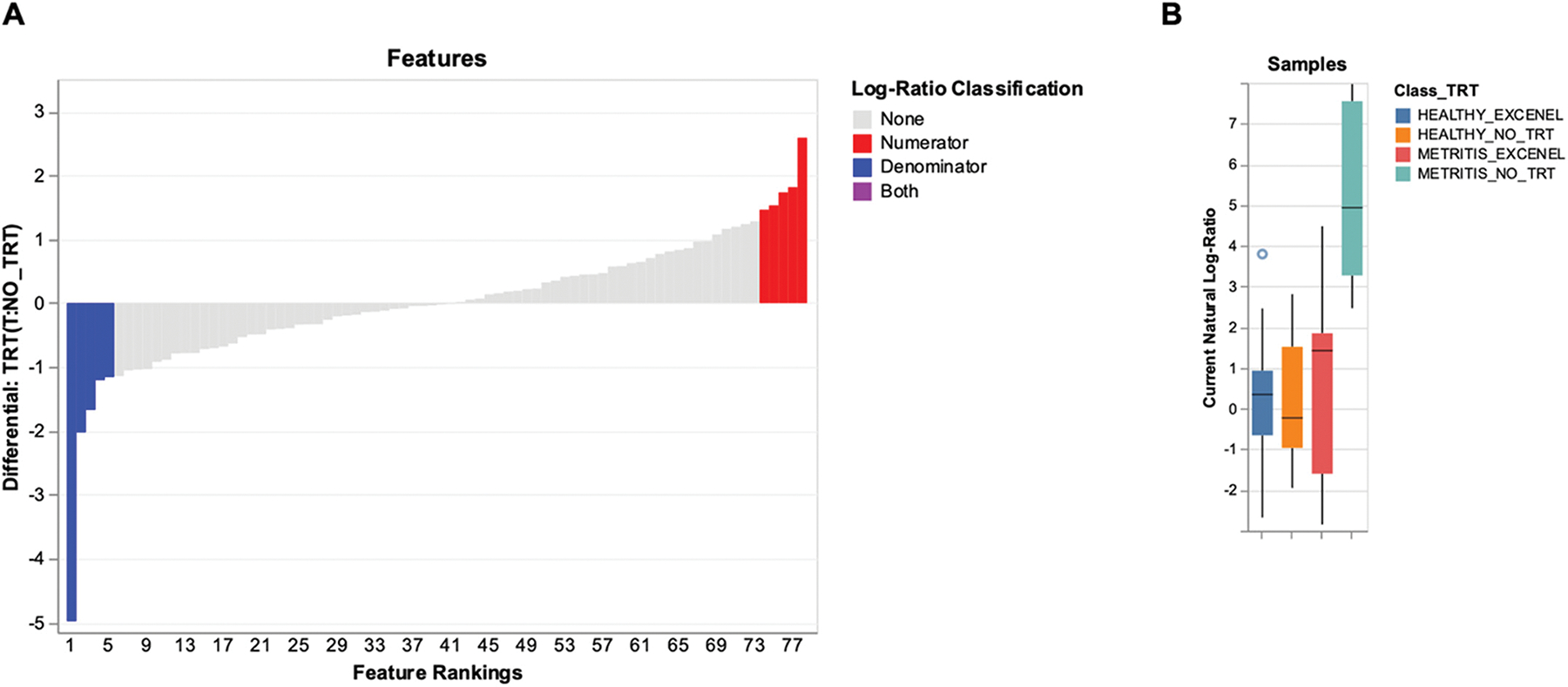
Multinomial regression analysis of antibiotic treatment effects on the uterine microbiota at 1 mo postpartum using the full Songbird model. (A) Rank plot illustrating the differential abundance of selected numerator features (more abundant in ceftiofur-treated cows) relative to denominator features (more abundant in untreated cows), with positive log-ratio values indicating enrichment of numerator features. The top 5 amplicon sequence variants (ASV) with increased (red) or decreased (blue) abundance are highlighted (see Supplemental Table S8), with the x-axis showing ranked features by differential abundance and the y-axis displaying log-ratio magnitudes. TRT(T:NO_TRT) denotes the differential log-fold change in microbial feature (e.g., ASV) abundance for antibiotic-treated cows (T) relative to untreated cows (NO_TRT), which serves as the reference level. Positive differential values indicate higher abundance in treated cows, while negative values indicate higher abundance in untreated cows. (B) Sample plot of log ratios (balances) of numerator to denominator features, based on Songbird differentials, across sample groups (healthy ceftiofur-treated [EXCENEL], healthy untreated [NO_TRT], metritis EXCENEL, and metritis NO_TRT). The sample plot highlights the abundance of the top 5 ASV increased in ceftiofur-treated cows (numerator) versus the top 5 ASV in untreated cows (denominator). The relative abundance of *Fusobacterium* was 145.45 times lower in ceftiofur-treated cows compared to untreated cows. The sample plot further demonstrates that ceftiofur-treated cows that had been diagnosed with metritis at 5 to 10 d postpartum exhibited a microbiome resembling that of healthy cows for these selected features.

The full Songbird model demonstrated superior predictive accuracy (Figure 5A) and was used to evaluate the persistent effects of ceftiofur treatment, administered 5 to 10 d postpartum, on the uterine microbiota at 1 mo postpartum (Figure 7). The top 5 features that increased or decreased with antibiotic treatment and the model’s differential (natural log of fold changes in taxon abundance between treated and untreated cows) are detailed in Supplemental Table S9 (see Notes). Fold changes were calculated (Fold change = e^differential^) to further demonstrate the significant shifts in microbial composition resulting from systemic antibiotic treatment. Notably, compared with ceftiofur-treated cows, untreated cows exhibited significantly greater abundances of key taxa, including *Fusobacterium* (145.45-fold), *Bacteroides vulgatus* (7.57-fold), an unclassified *Peptostreptococcaceae* (5.32-fold), an uncultured *Ruminococcaceae* bacterium (3.33-fold), and *Finegoldia* (3.18-fold). Conversely, ceftiofur-treated cows showed increased abundances of *Histophilus* (13.29-fold), *Candidatus mycoplasma* (6.13-fold), an uncultured *Streptobacillus* (5.66-fold), *Mycoplasma feliminutum* (4.61-fold), and *Anaerosalibacter* (4.31-fold). These taxon-specific shifts indicate long-term effects of ceftiofur treatment on selected microbiota, especially *Fusobacterium*, restoring the microbiota of metritis-treated cows toward that of healthy cows (treated or untreated), as illustrated in Figure 7B.

### Post Hoc Power Calculation

A detailed description of all power analyses is provided in Supplemental Appendix S1. Briefly, the study had high power (100%) to identify microbiota differences using 16S rRNA gene sequencing for cows with metritis and healthy cows at the time of diagnosis (5 to 10 d postpartum); high power (85%) to detect lasting effects of disease status (healthy vs. metritis) on the microbiota at 1 mo postpartum using 16S gene sequencing; and high power (100%) to detect differences between a clear and infected uterus at 1 mo postpartum using 16S gene sequencing. The study was underpowered to detect lasting effects of antibiotic treatment administered at disease diagnosis on the uterine microbiota composition at 1 mo postpartum (16S gene sequencing). The power of test for analyses of culture data (bacteriology) are presented as footnotes in the respective tables.

## DISCUSSION

This study evaluated the effects of ceftiofur treatment on the uterine microbiome and endometrial inflammation at 1 mo postpartum in cows diagnosed with metritis or classified as healthy at 5 to 10 d postpartum. The first objective was to determine whether ceftiofur treatment altered the uterine microbiome at 1 mo postpartum in these 2 groups of cows. With respect to microbiome, the cows that were initially assigned to this trial as either “metritis” or “healthy” were clearly different at 5 to 10 d postpartum. Our results align with results reported in recent reviews that summarized the uterine microbiota of metritis and healthy cows (Galvão et al., 2019a; Çömlekcioğlu et al., 2024). We found *Bacteroides*, *Fusobacterium*, and *Porphyromonas* as the dominant genera in metritis cows. The greater evenness and lesser diversity found in cows diagnosed with metritis typify the dysbiosis often described in acute infectious diseases such as metritis. When the broader microbial community was examined (phylogenetic tree by EMPress), the bulk of sequenced organisms were similarly represented in both healthy cows and cows with metritis. Only ~5% (28 out of 541) of the sequenced ASV at disease diagnosis were differentially abundant between metritis and healthy controls according to ANCOM. These findings support the idea that dysbiosis in metritis is mainly associated with the overgrowth of a small number of bacterial species.

The differences in the microbiota at disease diagnosis for metritis and healthy cows were still present at 1 mo postpartum based on both bacteriology and 16S RNA gene sequencing. Most studies conclude that the microbiome of cows initially diagnosed as metritis or healthy will converge over time (Galvão et al., 2019b; Figueiredo et al., 2024), but the extent to which it does may depend on the timing and method of collection (i.e., uterine lavage, vaginal sample). We found in a similar study in which the microbiome was sampled from a uterine lumen of a first-parity cow after slaughter that there were minimal differences in the microbiome at 80 to 165 d postpartum for metritis and healthy cows (Silva et al., 2024). We also performed bacteriology on the uterine body and horns from cows in the same study but either failed to isolate bacteria or only isolated a small number of bacteria from most cows. In this study, the microbiome was clearly different at 1 mo postpartum for cows that were previously diagnosed as metritis or healthy. We found no evidence that bacterial populations (either cultured [data not shown] or sequenced) differed for the gravid and nongravid uterine horns. Perhaps the connection of horns through the uterine body allows microbial migration from one horn to another.

Based on bacterial culture, the bacteria that were found in the postpartum uterus at 5 to 10 d postpartum were still present at 1 mo postpartum, particularly in cows with purulent flush or an acute metritis-like infection. Approximately 50% of the cows that were originally diagnosed as healthy had no growth for the bacteriology. This percentage was lower (17%) for the cows originally diagnosed with metritis. Typical metritis-associated bacteria, including *Fusobacterium necrophorum*, *Trueperella pyogenes*, and *Helcococcus ovis*, were cultured 1 mo postpartum. *Escherichia coli* is commonly associated with metritis (Carneiro et al., 2016), but we only cultured 3 *Escherichia coli* colonies from the uterine lumen of a single cow (metritis cow with purulent lumen). At the same time, *Escherichia coli* was cultured from the external surface of the uterus from 15 cows with an average of 21 colonies per sample. The observed infrequent culture of *Escherichia coli* within the uterine lumen might be explained by the methods we used to sample the uterine lumen in this study (reproductive tract collected at slaughter and sampled within a biosafety cabinet), which would have reduced environmental contamination.

One of the most important factors that determine the uterine microbiome at 1 mo postpartum was whether the cow had purulent material in the uterus at slaughter. Purulent or acute uterine infections were observed at slaughter 1 mo postpartum in 29% of cows initially deemed healthy and in 56% of cows initially diagnosed with metritis. In their analyses of risk factors for subclinical endometritis (**SCE**) in 39 commercial dairy herds in New York State, Cheong et al. (2011) reported a prevalence of 25.9% for SCE, with acute metritis increasing the odds ratio for SCE by 2 fold (Cheong et al., 2011). These findings are consistent with our results, where roughly one-quarter of healthy cows and twice as many metritis cows had purulent material at slaughter. In this study, cows diagnosed as metritis or healthy differed for their microbiome at 1 mo postpartum but the reclassification of cows based on the content of the uterine lumen (clear, purulent, or acutely infected) created a more distinct separation of groups for the bacterial culture data as well as 16S rRNA gene sequencing. When samples were analyzed to investigate the influence of flush phenotype (clear, purulent, acute infection), there were a greater number of sequence reads for the cows with a purulent or acutely infected uterus. The 3 distinct flush phenotypes clustered separately on the Bray–Curtis PCoA plot. Additionally, cows with infected uteri (purulent + acute) exhibited lower evenness and reduced β-diversity in their uterine microbiota compared with cows with a clear uterine flush at 1 mo postpartum. This is similar to the results by Pascottini et al., (2020) that reported less bacterial diversity in the uterus of cows with clinical endometritis compared with healthy cows.

According to the results of the multinomial regression analysis conducted using Songbird (Morton et al., 2019), an acute infection and a purulent lumen were defined by an entirely different microbiome. Acute infection, for example, was defined by dominant genera and species such as *Campylobacter*, *Gallicola*, *Trueperella*, *Anaerosalibacter*, *Peptostreptococcus*, *Clostridiales bacterium*, *Bacteroidales bacterium*, and *Bacteroides.* These species are well-established metritis pathogens (Silva et al., 2024), and many were also shown to be differentially expressed at disease diagnosis in the present study. Conversely, cows with a purulent lumen were defined by *Caviibacter*, *Candidatus Mycoplasma*, *Ureaplasma diversum*, *Mycoplasmopsis bovigenitalium*, *Fusobacterium necrophorum*, *Streptobacillus*, *Actinomyces*, *Campylobacter*, and *Porphyromonas levii*, bacterial genera and species that are known to populate the uterus, some of which are also associated with metritis. Interestingly, *Ureaplasma diversum*, associated with a healthy uterus at disease diagnosis, was later increased in the uterine lumen of cows with a purulent uterine flush 1 mo postpartum. Cows with a clear uterine flush exhibited a more diverse microbiome, including microbes associated with a healthy uterus, such as *Cutibacterium* and *Mycoplasma wenyonii*, as well as microbes linked to metritis, such as *Trueperella, Porphyromonas asaccharolytica,* and *Helcococcus ovis*. This greater microbial diversity may have played a protective role by preventing dysbiosis and mitigating the pathogenic effects of the present bacteria, ultimately reducing the risk of disease.

Given the differences in the uterine microbiome at 1 mo postpartum for cows based on their initial diagnosis (healthy or metritis) and their eventual uterine flush classification (clear, purulent or acute), the next question we examined was whether the antibiotic treatment affected the microbiome at 1 mo postpartum. The effects of antibiotic treatment on cultured species was detected as a treatment by status (healthy or metritis) interaction for the total number of colonies we isolated from the uterine lumen. In healthy cows, antibiotic treatment did not change the number of colonies isolated from the uterine lumen at 1 mo postpartum. Conversely, in cows with metritis, antibiotic treatment reduced the total number of colonies that we isolated. Our interpretation is that treating a cow with ceftiofur effectively at disease diagnosis reduces the bacterial load (measured as the number of colonies formed in bacterial culture) at 1 mo postpartum. The effect of antibiotic on the total number of colonies in metritis cows should be considered preliminary given the relatively low power of the test (60%). We did notice an effect of antibiotic on the prevalence of the most common bacteria in cows with a purulent or acute lumen (reduced prevalence in treated cows). This result also should be considered preliminary given the relatively low power of the test (60%). Regarding the effects of antimicrobial treatment on the 16S rRNA sequencing results, treatment 5 to 10 d postpartum decreased the number of sequencing reads and β-diversity of the uterine microbiome 1 mo postpartum irrespective of the initial disease diagnosis. When the multinomial regression analysis was employed to investigate the effects of antimicrobial treatment on the uterine microbiome 1 mo postpartum, we observed that antibiotic treatment had a major targeted effect on the genera *Fusobacterium*. The observed effect of ceftiofur on *Fusobacterium* is consistent with previous research demonstrating that systemic ceftiofur administration rapidly reduces (within 2 to 5 d) the abundance of *Fusobacterium* (Jeon et al., 2018, 2021).

The second objective was to determine if antibiotic treatment at 5 to 10 d postpartum had an effect on endometrial inflammation at 1 mo postpartum. The null hypothesis was that antibiotic-treated cows (either healthy or metritis) would have inflammation scores similar to untreated cows at 1 mo postpartum. If true, then this lack of an effect could explain why ceftiofur treatment may increase clinical cure early postpartum but fails to increase pregnancy risk, as ceftiofur does not have a lasting effect on uterine inflammation. Large clinical trials of the effects of metritis and endometritis typically classify cows as either healthy, subclinical endometritis (>5% neutrophils in cytology sample), or clinical endometritis (>5% neutrophils in cytology sample with purulent vaginal discharge). This study assessed uterine disease by observing the contents of the uterine lumen. Others (Pascottini et al., 2020) have shown that the microbiota differs between cows with clinical endometritis and clinically healthy cows, but is similar between cows with subclinical endometritis and healthy cows. A cytobrush exam was not conducted for this study, so we cannot say whether the cows that had a clear uterine flush could be further subdivided into cows with subclinical endometritis versus healthy cows. The histological analysis determined that the flush phenotype (clear versus purulent or acutely infected) was more closely aligned with the measures of inflammation when compared with the original disease diagnosis (metritis or healthy). The only exception was eosinophil score, where metritis cows had a greater score than healthy cows. There were 5 cows (29%) originally diagnosed as healthy that were found to have purulent material in the uterus at 1 mo postpartum. Likewise, 8 cows (44%) that had been previously diagnosed with metritis presented a clear uterine lumen at 1 mo postpartum. Notably, 5 out of 8 cows in the metritis clear flush group had retained placenta after calving, indicating that the residual placental tissue had been entirely broken down and either expelled or resorbed. The strong association between a purulent lumen and endometrial inflammation and the existence of cows that switched from healthy (d 5 to 10) to diseased (1 mo) and vice versa precluded the association between original disease diagnosis (healthy or metritis) and inflammation at 1 mo postpartum.

Overall, antibiotic treatment had a small but consistent effect on the inflammation at 1 mo postpartum. Specifically, antibiotic treatment reduced epithelial, stromal, and caruncular inflammation score and eosinophil score in healthy cows but increased the same inflammation scores in metritis cows. We also found that eosinophil score at 1 mo postpartum was greater in metritis compared with healthy cows. We anticipated that antibiotic treatment would reduce inflammation by killing bacteria, speeding the resolution of disease, but this was not the case in metritis cows. With respect to early postpartum inflammation, both living and dead bacteria have the capacity to cause inflammation via the release of damage-associated molecular patterns as well as pathogen-associated molecular patterns (Sheldon et al., 2019). Unresolved inflammation that is associated with uterine disease is likely arising from both living and nonliving bacteria that are degraded, broken down, resorbed into the uterine wall, or expelled through the cervical opening. Perhaps the increase in inflammatory score in antibiotic-treated metritis cows demonstrates a greater inflammatory response occurring during disease resolution in metritis cows. This does not explain, of course, why the inflammatory score decreased in antibiotic-treated healthy cows.

Although fertility was not evaluated in the current study, previous work demonstrated that the presence of a purulent or acutely infected uterus 1 mo postpartum was associated with reduced conception rates and increased pregnancy loss following first breeding (Bromfield et al., 2015; Moraes et al., 2021; LeBlanc, 2023). Although the mechanisms by which metritis and endometritis contribute to infertility and how the microbiota interacts with normal uterine tissue regeneration and repair in cows are not yet fully understood, uterine disease appears to leave a lasting imprint on endometrial gene expression, particularly in the caruncular endometrium, potentially impairing placentation (Silva et al., 2024). In addition to reducing uterine competence, postpartum uterine disease negatively affects ovarian function, oocyte competence, and preimplantation embryo development (Ribeiro et al., 2016; Caldeira et al., 2025a). The contents of the uterine lumen at 1 mo postpartum (clear versus purulent or acute) were associated with measures of inflammation in the uterine lumen. It is likely that this inflammation within the diseased uterus leaves a lasting imprint on the function of uterine cells through mechanisms involving inflammatory memory (Ordovas-Montanes et al., 2020). A deeper understanding of the pathogenicity and host-microbiota interactions related to uterine disease, as well as their downstream effects on uterine and ovarian tissues, is essential for developing effective strategies to reduce the occurrence and impact of postpartum uterine diseases on dairy cow fertility.

## CONCLUSIONS

The principal findings were that the uterine microbiome at 1 mo postpartum differed for cows diagnosed as metritis or healthy at 5 to 10 d postpartum. Approximately 40% of cows had a purulent or acute-infected uterus a slaughter. There was a modest effect of early postpartum antibiotic treatment on the uterine microbiome at 1 mo postpartum, and this treatment appeared to specifically target *Fusobacterium*. Endometrial inflammation was primarily linked to the contents of the uterine lumen (greater in cows with a purulent or acutely infected uterus). Antibiotic (ceftiofur) treatment had a small effect on uterine inflammation but paradoxically increased inflammation score relative to untreated control. Future research should elucidate the biological mechanisms driving clinical cure of metritis and its relationship with fertility postpartum.

## Figures and Tables

**Table 1.** Number of cows classified as either healthy or diagnosed with metritis, treated with antibiotics (Ab), or not treated (No trt) at 5 to 10 d postpartum^1^

Flush	Healthy (n = 17)			Metritis (n = 18)^2^		
	Ab (n = 9)	No trt (n = 8)	Total (%)	Ab (n = 9)	No trt (n = 9)	Total (%)

Clear	6	6	12 (71)	4	4	8 (44)
Purulent	3	2	5 (29)	4	3	7 (39)
Acute infection	0	0	0	1	2	3 (17)
Total	9	8	17 (100)	9	9	18 (100)

1 Cows were subsequently categorized based on uterine condition at slaughter (1 mo postpartum) into one of the following groups: clear uterine lumen, purulent uterine lumen, or acute uterine infection resembling early postpartum metritis.

2 The metritis group included 11 cows with a diagnosis of retained placenta (failure to pass the placenta out of the uterine lumen with 24 h after calving). The number of cows with retained placenta in each group was as follows: 5 out of 8 cows in the clear flush group (2 Ab and 3 no trt), 4 out of 7 cows in the purulent group (2 Ab and 2 no trt), and 2 out of 3 cows in the acute group (1 Ab and 1 no trt).

**Table 2.** Least squares means and SE for fibrosis, epithelium inflammation, stromal inflammation, and eosinophil score derived from a histological evaluation of uterine cross sections; data are presented for healthy versus metritis cows that were either treated with antibiotic (Ab) or not treated (No trt) with antibiotic and clear versus purulent or acutely infected uterine flush that were either Ab treated or not treated at disease diagnosis

Item^1^	Healthy (n = 17)			Metritis (n = 18)			Clear (n = 20)			Purulent or acutely infected (n = 15)		
	Ab (n = 9)	No trt (n = 8)	SE	Ab (n = 9)	No trt (n = 9)	SE	Ab (n = 10)	No trt (n = 10)	SE	Ab (n = 8)	No trt (n = 7)	SE

Fibrosis score	2.45	2.56	0.11	2.53	2.57	0.09	2.31	2.45	0.09	2.67	2.67	0.12
Epithelial inflammation score^x^	0.91	1.08	0.08	1.22	0.97	0.09	0.89	0.75	0.05	1.24	1.29	0.12
Stromal inflammation score^x^	2.60	2.75	0.04	2.74	2.66	0.04	2.66	2.61	0.03	2.68	2.80	0.04
Caruncular inflammation score^x^	2.88	2.94	0.02	2.96	2.91	0.02	2.90	2.89	0.02	2.93	2.96	0.02
Eosinophil score**^,x^	0.98	1.25	0.11	1.77	1.39	0.16	1.03	0.81	0.13	1.71	1.83	0.15

x Superscript letter indicates the interaction of the main effect with treatment (^x^*P* < 0.05).

1 Histological scoring of endometrial fibrosis (1 to 3; mild to severe) and endometrial inflammatory cell or eosinophil scored from 0.5 (<10 cells/field) to 3 (>50 cells/field). Retrospective power of test for main effect: eosinophil score, 100%. Retrospective power of test for main effect by treatment: epithelial inflammation score, 96%; stromal inflammation score: 100%; caruncular endometrium score, 79%; eosinophil score: 100%. Retrospective power of test for main effect: fibrosis score, 99%; epithelial inflammation score, 100%; stromal inflammation score, 100%; caruncular inflammation score, 67%; eosinophil score, 100%. Retrospective power of test for main effect by treatment: stromal inflammation score: 99%.

** *P* < 0.01, for the probability of the main effect (healthy versus metritis or clear versus purulent or acutely infected).

**Table 3.** Prevalence of cultured bacterial species isolated from the lumen of the uterine horn following slaughter at 1 mo postpartum for cows that were either healthy or metritis and treated with antibiotic (Ab) or not treated (No trt) at 5 to 10 d postpartum; the list includes species isolated from at least 3 cows

Species^1^	Healthy (n = 17)			Metritis (n = 18)		
	Ab (n = 9)	No trt (n = 8)	Total (%)	Ab (n = 9)	No trt (n = 9)	Total (%)

*Bacteroides pyogenes*	0	1	1 (6)	1	1	2 (11)
*Corynebacterium renale*	0	0	0 (0)	1	2	3 (17)
*Cutibacterium acnes*	1	0	1 (6)	1	1	2 (11)
*Fusobacterium necrophorum*	0	0	0 (0)	3	4	7 (39)
*Helcococcus ovis*	0	0	0 (0)	2	1	3 (17)
*Histophilus somni*	1	0	1 (6)	1	7	8 (44)
*Paenibacillus cookii*	0	1	1 (6)	1	1	2 (11)
*Peptoniphilus indolicus*	0	0	0 (0)	0	4	4 (22)
*Porphyromonas levii*	0	0	0 (0)	2	3	5 (28)
*Prevotella heparinolytica*	0	0	0 (0)	1	2	3 (17)
*Staphylococcus epidermidis*	0	1	1 (6)	2	0	2 (11)
*Streptococcus pluranimalium*	0	0	0 (0)	2	2	4 (22)
*Trueperella pyogenes*	1	0	1 (6)	4	5	9 (50)
No growth	3	5	8 (47)	2	1	3 (17)

1 Across all species, greater prevalence for metritis versus healthy (*P* < 0.001) and no effect of antibiotic treatment (*P* = 0.148) or antibiotic by group interaction (*P* = 0.174). Retrospective power of test for main effect (heathy vs. metritis): prevalence, 100%.

**Table 4.** Partial list of cultured bacterial species isolated from the lumen of the uterine horn following slaughter at 1 mo postpartum for cows that had either a clear uterine lumen or a purulent or acute uterine lumen at slaughter and previously treated with antibiotic (Ab) or not treated (No trt) at 5 to 10 d postpartum; the list includes species isolated from at least 3 cows

Species^1^	Clear (n = 20)			Purulent + acute (n = 15)		
	Ab (n = 10)	No trt (n = 10)	Total (%)	Ab (n = 8)	No trt (n = 7)	Total (%)

*Bacteroides pyogenes*	0	0	0 (0)	1	2	3 (20)
*Corynebacterium renale*	1	1	2 (10)	0	1	1 (7)
*Cutibacterium acnes*	1	1	2 (10)	0	1	1 (7)
*Fusobacterium necrophorum*	0	0	0 (0)	3	4	7 (47)
*Helcococcus ovis*	0	0	0 (0)	2	1	3 (20)
*Histophilus somni*	0	3	3 (15)	2	4	6 (40)
*Paenibacillus cookii*	0	1	1 (5)	1	1	2 (13)
*Peptoniphilus indolicus*	0	0	0 (0)	0	4	4 (27)
*Porphyromonas levii*	0	0	0 (0)	2	3	5 (33)
*Prevotella heparinolytica*	0	0	0 (0)	1	2	3 (20)
*Staphylococcus epidermidis*	2	1	3 (15)	0	0	0 (0)
*Streptococcus pluranimalium*	1	0	1 (5)	1	2	3 (20)
*Trueperella pyogenes*	2	0	2 (10)	3	5	8 (53)
No growth	3	6	9 (45)	0	2	2 (13)

1 Across all species, greater prevalence for purulent + acute versus clear (*P* < 0.001) and lesser prevalence in antibiotic-treated purulent + acute cows (antibiotic by group interaction; *P* = 0.0312). Retrospective power of test for main effect (clear vs. purulent + acute): prevalence, 100%. Retrospective power of test for main effect by treatment: prevalence, 60%.

**Table 5.** Median (25th and 75th percentile) number of bacterial species and total colonies isolated from uterine lumen at 1 mo postpartum, showing the influence of antibiotic treatment on the microbiome of cows previously diagnosed with or without metritis (treated at 5 to 10 d postpartum) and with the distinct uterine flush phenotypes (clear vs. purulent + acute) at slaughter

Status/flush	Samples from the uterine lumen (gravid and nongravid horn combined)							
	No. of cows	Median (25th, 75th percentile)				Statistical analysis (*P* <)^1^		
		No. of species		Total no. of colonies		Effect Status	Species 0.0001	Colonies 0.0001
		Antibiotic	No treatment	Antibiotic	No treatment			

Healthy	17	1 (0, 2)	0 (0, 1)	1 (0, 3)	0 (0, 2)	Flush	0.0302	0.0886
Metritis	18	3 (2, 8)	9 (6, 12)	11 (2, 601)	1,516 (1,005, 1,923)	Trt	0.4316	0.1488
Clear	20	1.5 (0, 2)	0 (0, 3)	1.5 (0, 51)	0 (0, 1,000)	Trt × status	0.2250	0.0306
Purulent + acute	15	3 (1, 7.5)	11 (1, 12)	7 (1.5, 634)	1,664 (6, 2,080)	Trt × flush	0.6275	0.5986

1 Results of statistical analysis that included the effects of status (healthy vs. metritis), flush (clear vs. purulent + acute), and treatment (Trt; antibiotic vs. no treatment). Retrospective power of test for status (healthy vs. metritis): number of species, 100%, total number of colonies, 99%. Retrospective power of test for flush (clear vs. purulent + acute): number of species, 60%, total number of colonies, 40%. Retrospective power of test for treatment (antibiotic vs. no treatment) by flush (clear vs. purulent + acute): total number of colonies, 59%.

## Nonstandard abbreviations used:

Ab: treated with antibiotics
ANCOM: analysis of composition of microbes
ASV: amplicon sequence variant
BHI: brain-heart infusion
clr: centered log ratio
EXCENEL: ceftiofur-treated
EXT: external surface of the uterus
IQR: interquartile range
NCBI: National Center for Biotechnology Information
No trt: not treated; NO_TRT = untreated
PCoA: principal coordinate analysis
PEA: phenylethyl alcohol agar
PERMANOVA: permutational multivariate ANOVA
SCE: subclinical endometritis
TSA: tryptic soy agar with 5% sheep blood.
